# Transcriptomics Analysis Reveals New Insights into the Roles of Notch1 Signaling on Macrophage Polarization

**DOI:** 10.1038/s41598-019-44266-4

**Published:** 2019-05-29

**Authors:** Chetan P. Hans, Neekun Sharma, Sidharth Sen, Shuai Zeng, Rishabh Dev, Yuexu Jiang, Advitiya Mahajan, Trupti Joshi

**Affiliations:** 1 Department of Cardiovascular Medicine, University of Missouri, Columbia, USA; 2 Medical Pharmacology and Physiology, University of Missouri, Columbia, USA; 3 Dalton Cardiovascular Research Center, University of Missouri, Columbia, USA; 4 MU Informatics Institute, University of Missouri, Columbia, USA; 5 Department of Electrical Engineering and Computer Science, University of Missouri, Columbia, USA; 6 Department of Health Management and Informatics, School of Medicine, University of Missouri, Columbia, USA; 7 Christopher S. Bond Life Science Center, University of Missouri, Columbia, USA

**Keywords:** Cytokines, Interleukins

## Abstract

Naïve macrophages (Mφ) polarize in response to various environmental cues to a spectrum of cells that have distinct biological functions. The extreme ends of the spectrum are classified as M1 and M2 macrophages. Previously, we demonstrated that Notch1 deficiency promotes Tgf-β2 dependent M2-polarization in a mouse model of abdominal aortic aneurysm. The present studies aimed to characterize the unique set of genes regulated by Notch1 signaling in macrophage polarization. Bone marrow derived macrophages isolated from *WT* or *Notch1*^+/−^ mice (n = 12) were differentiated to Mφ, M1 or M2-phenotypes by 24 h exposure to vehicle, LPS/IFN-γ or IL4/IL13 respectively and total RNA was subjected to RNA-Sequencing (n = 3). Bioinformatics analyses demonstrated that *Notch1* haploinsufficiency downregulated the expression of 262 genes at baseline level, 307 genes with LPS/IFN-γ and 254 genes with IL4/IL13 treatment. Among these, the most unique genes downregulated by *Notch1 haploinsufficiency* included *fibromodulin* (*Fmod*), *caspase-4*, *Has1*, *Col1a1*, *Alpl* and *Igf*. Pathway analysis demonstrated that extracellular matrix, macrophage polarization and osteogenesis were the major pathways affected by *Notch1* haploinsufficiency. Gain and loss-of-function studies established a strong correlation between *Notch1* haploinsufficiency and *Fmod* in regulating Tgf-β signaling. Collectively, our studies suggest that *Notch1* haploinsufficiency increases M2 polarization through these newly identified genes.

## Introduction

Vascular diseases including abdominal aortic aneurysm (AAA), atherosclerosis, obesity and cancer present a group of highly prevalent chronic conditions that share common inflammatory pathways^1^. These diseases are characterized by acute or chronic inflammation that can affect a specific organ or the whole system^2–4^. The onset of inflammation in these conditions correlates with an alteration in the dynamic balance of pro-inflammatory vs. anti-inflammatory macrophages that play a decisive role in the initiation and perpetuation of disease^5–7^. Given their critical roles in disease pathogenesis, an understanding of the key factors that modulate macrophage differentiation may have tremendous therapeutic potential.

Naïve macrophages (Mφ), in response to the local milieu, differentiates towards a variety of polarized states with distinct functions^5^. The extreme ends of this spectrum of polarization are referred to as either pro-inflammatory (M1) or immune-regulatory (M2) macrophages. These macrophage populations are heterogeneous and opposing in terms of expression of surface markers and functions^5^. In response to a local injury, M1-polarized macrophages induce pro-inflammatory cytokines and chemokines^8,9^. The M2-phenotype on the contrary, diminishes the inflammatory response, promotes tissue repair and healing via production of collagen and elastin precursors^10^. Although, there have been extensive studies on the pro-inflammatory and anti-inflammatory cytokines associated with M1 or M2-macrophages, the key factors that modulate M1/M2 polarization have not been well-documented^11–14^. Recent studies have further sub-classified M2 macrophages into M2a, M2b, M2c or tumor-associated (TAMs) subsets depending upon the external stimulus and transcriptional changes^12,15,16^. Thus, the mechanism of macrophage polarization is exceedingly becoming complex and high-throughput studies are required to identify the key regulators for such polarization^13^.

We, and others, have shown that Notch1 signaling plays a causal role in M1/M2 differentiation during the development of several vascular pathologies^17–24^. The Notch signaling pathway consists of a family of four Notch receptors (1–4). Interactions with the Jagged and Delta ligand families results in the release and translocation of the Notch1 intracellular domain (NICD) into the nucleus where it binds and associates with the transcription factor Cp-binding factor-1 (CBF1; also known as Rbp-Jκ or Csl)^25–27^. Although, the current findings suggest a possible link between Notch activation and an inflammatory environment in many disease states, the possible interplay between Notch1 signaling and inflammation in the context of macrophage polarization is poorly studied^18–20,24,28,29^. We previously reported that inhibition of Notch signaling attenuates the progression of AAA at early stages by reducing the inflammatory response associated with a Tgf-β2 dependent increase in differentiation of M2-macrophages^20,21^. However, the molecular mechanism by which Notch1 signaling regulates macrophage polarization is unknown.

Using RNA sequencing (RNA-Seq), we aimed to: i) determine putative markers specific for M1 and M2 polarization and ii) analyze differentially expressed genes (DEGs) which are unique to *Notch1* haploinsufficiency. Our data revealed that *Notch1* haploinsufficiency decreases the expression of fibromodulin (Fmod), a cytosolic protein implicated in the M2-polarization of macrophages.

## Results

### Hierarchal clustering, correlation matrix and gene module prediction

**Figure 1:**
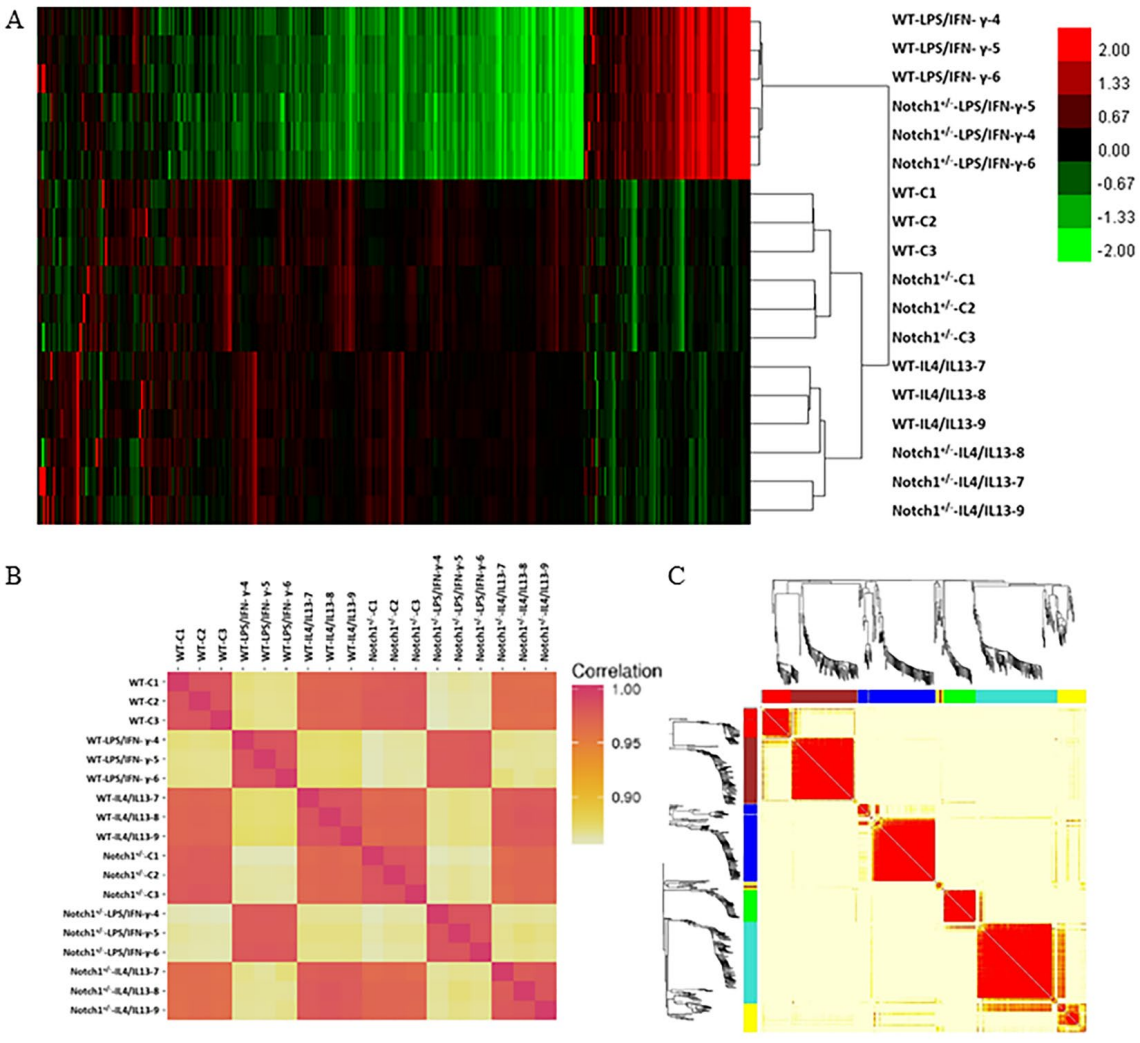
Data matrix analyses distributed the data into expected subgroups and treatments. (**A**) Heat map from 20,375 detectable mouse genes in *WT* and *Notch1*^+/−^ BMDMs treated with vehicle, LPS/IFN-γ or IL4/IL13. Reads per kilobase of transcript per million (RPKM) were log^2^-transformed and loaded into Gene Cluster 3.0. Each color-bar unit represents a difference of one log^2^ unit in RPKM. The green and red color indicates downregulation and upregulation of the genes respectively and the intensity of the color corresponds to fold difference in the expression. (**B**) Pearson’s correlation coefficient, calculated for all the 18 samples. (**C**) Weighted correlation network analysis (WGCNA) to visualize the correlation among the differentially expressed genes. The expression of genes with fold change value between -1 to 1 and q-value less than 0.05 were selected as inputs of WGCNA. The modules were constructed for this network by using hierarchical clustering. The genes with high correlation were clustered into different modules. With this threshold, the number of differential genes in *Notch1*^+*/–*^C vs. WT-C, in *Notch1*^+*/–*^IL4/IL13 versus WT-IL4/IL13 and in *Notch1*^+*/–*^LPS/IFN-γ versus WT-LPS/IFN-γ, were 199, 155 and 158 respectively.

The aim of the study was to determine the effect of *Notch1* haploinsufficiency on expression of genes that are unique to M1 or M2 phenotype, using the unbiased RNA-Seq approach. Bone marrow derived macrophages (BMDMs) were stimulated with lipopolysaccharide (LPS; 100 ng/ml) and interferon-γ (IFN-γ; 20 ng/ml) or IL-4/IL-13 (10 ng/ml each) for 24 h to polarize into M1 or M2 phenotype respectively. Reads per kilobase of transcript, per million mapped reads (RPKM) data for detectable mouse genes (>RQT in at least one sample) was used for hierarchical clustering analysis by Cluster 3.0 software^30^. Genes were median centered prior to hierarchical clustering and analysis was conducted using centered correlation as the similarity metric and average linkage as the clustering method (Fig. 1A). Heat maps of all the expressed genes demonstrated that the naïve macrophages (Mφ), LPS/IFN-γ treated or IL4/IL13 treated BMDMs from WT mice clustered most closely with respective treatments to the *Notch1*^+/−^ BMDMs (Fig. 1A). LPS/IFN-γ treated macrophages clustered farthest away from the IL4/IL13 treated macrophages suggesting that these stimulants had different and contrasting effects. Pearson’s correlation coefficient analysis for all 18 samples showed strong correlation between *WT* and *Notch1*^+/−^ BMDMs at baseline levels or with similar treatments (Fig. 1B).

Gene co-expression networks were determined using weighted correlation network analysis (WGCNA)^31^, to compute and visualize the correlation among a list of differentially expressed genes (Fig. 1C and Table 1). WGCNA gene module analysis on combined differential gene list for WT and *Notch1*^+/−^ datasets for all three conditions predicted 7 gene modules (Fig. 1C). The different gene modules have 35 (red), 83 (brown), 93 (blue), 8 (grey), 39 (green), 100 (turquoise) and 41 (yellow) genes in each module. For the brown module, the predicted pathways having at least 3 mapped genes included metabolic, Ras and Rap1 signaling pathways. For the blue module, the predicted pathways included PI3K-Akt signaling, focal adhesions, ECM-receptor interactions and cell adhesion molecules. For the green module, pathways included calcium signaling, oocyte meiosis, and the dopaminergic synapse. For the turquoise module, cell adhesion molecules (CAMs), PI3K-Akt signaling, leukocyte trans-endothelial migration, Rap1 signaling, antigen processing and presentation pathways were implicated with more than 3 genes mapped. The yellow module had more than three genes mapped to metabolic pathways, ECM-receptor interactions and proteoglycans in cancer. Red and grey modules did not have genes that have known pathway mappings. Using KBCommons database and in-house novel algorithm, we performed a step-wise active pathway detection using a dynamic programming approach. The results are represented as tree structures showing the summarized networks for the control, LPS/IFN-γ and IL4/IL13 treatments respectively with Notch1 as the root of the tree (Supplemental Fig. 1A–C). Among these three tree comparisons as shown in Venn diagram (Supplemental Fig. 1D) 12 genes were common to all of them (*Myc*, *Fgr*, *Ptk2*, *Pik3cb*, *Rbpjl*, *Hspa8*, *Hkdc1*, *Glb1*, *Vegfc*, *Akt3*, Mmp9 and *Notch1*). There are 14 additional genes common between the control and LPS/IFN-γ tree (*Ldha*, *Prkcd*, *Mmp14*, *Ngfr*, *Pkm*, *Eif4ebp2*, *Mapk3*, *Dlat*, *Pdhb*, *Mtor*, *Mapk12*, *St3gal2*, *St6galnac6*, and *Plau*), while 7 genes common between LPS/IFN-γ and IL4/IL13 (*Apaf1*, *Casp4*, *Sdc1*, *Itgb3*, *Col1a2*, *Trp53*, and *Col1a1*). There were 6 common genes between Control and IL4/IL13 treatment (*Pdpk1*, *Foxo3*, *B4galt2*, *B3galt1 and St3gal4*, and *Mmp2*). Control has 45 and LPS/IFN-γ has 58 unique genes in the tree, while IL4/IL13 tree has 53 unique genes as shown (Supplemental Fig. 2D). The visualization of these graphical data matrix depict that the variance in the data set are primarily driven by different treatments and the divergence of the pattern of these data seems to be characteristic for each sample group.

**Table 1:** Pathway Analysis Summary. Criteria selected based on the Tukey *p* value (<0.05) and Fold Change (>2 up or down) for the specified comparison

Description	Total Input IDs	WebGestalt Mapped IDs	Unique IDs	KEGG Results	WIKI Results	GO categories		
						Biological Process	Molecular Function	Cellular Component
Reference Gene Set	20375	12413	12410	NA				
Notch1^+/−^ vs WT-C FC >1.5 Up	260	148	148	4 (4)	6 (5)	40 (40)	23 (8)	33 (25)
Notch1^+/−^ vs WT-(C) FC >1.5 Down	433	383	383	9 (8)	13 (11)	40 (40)	40 (40)	40 (40)
Notch1^+/−^ vs WT (LPS/IFN-γ) FC >1.5 Up	234	142	142	29 (23)	12 (11)	40 (40)	40 (30)	21 (18)
Notch1^+/−^ vsWT (LPS/IFN-γ) FC >1.5 Down	763	599	599	8 (8)	6 (5)	40 (40)	40 (40)	40 (40)
Notch1^+/−^ vs WT (IL4/IL13) FC >1.5 Up	99	48	48	4 (2)	7 (6)	40 (40)	15 (13)	12 (12)
Notch1^+/−^ vs WT (IL4/IL13) FC > 1.5 Down	476	402	402	13 (7)	5 (2)	40 (40)	40 (40)	40 (40)

Principal Component Analysis (PCA) was performed on the RPKM data for 17,400 low p-value (minimum ANOVA *p* < 0.05) mouse genes. The RPKM values were log10-transformed, centered so that each sample had mean 0, and the first three principal components were calculated (Supplemental Fig. 2). To find genes that substantially contribute to each PCA value, the correlation and fold-change in expression of each gene with the first three principal components was calculated according to the methods of Sharov *et al*.^32^. Genes with a positive or negative correlation of at least 0.9 and a fold change of at least 2.0 compared with the principal components are reported (Supplemental Fig. 2). The close clustering of the groups with similar treatment as revealed by PCA suggests relatedness of samples and that the alterations in the expression of genes among the dataset can be attributed to variance induced by *Notch1 haploinsufficiency*, thus validating the approach.

### Notch1 haploinsufficiency differentially regulates novel M1/M2 genes at baseline

**Figure 2:**
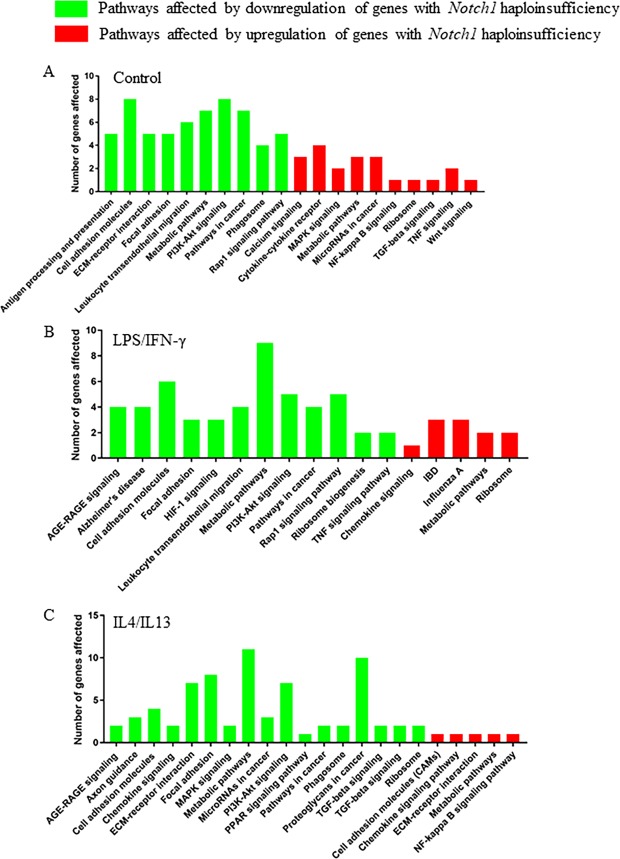
*Notch1* haploinsufficiency affects numerous pathways related to macrophage polarization and cytokine/chemokine signaling. Pathway analysis showing the major pathways affected by the downregulation (green) or upregulation (red) of genes by *Notch1* haploinsufficiency in naïve macrophages (**A**), in response to LPS/IFN-γ (**B**) or IL4/IL13 (**C**). ‘Y’ axis display the number of genes implicated in each pathway.

*Notch1* haploinsufficiency downregulated the expression of ~250 genes below 50% of the WT Mφ in the absence of external stimulant (Table 2; left green panel). Among this category, the prominent genes were hyaluronan synthase 1 (*Has1*), caspase 4 (*Casp4*), fibromodulin (*Fmod*), hairy/enhancer-of-split related with YRPW motif-like (*HeyL*), hairy and enhancer of split 1 (*Hes1*), delta-like 4 (*Dll4*), collagen, type I, alpha 1 (*Col1a1*), and insulin-like growth factor 2 (*Igf2*). Categorization of these genes to Gene Ontology (GO)^33^ and KEGG pathway^34^ revealed that common pathways affected by *Notch1* haploinsufficiency at baseline levels include leukocyte trans-endothelial migration, cell adhesion molecules, ECM-receptor interactions, focal adhesion, cancer, RAS signaling, cAMP signaling, Rap1 signaling, Pi3k-Akt signaling and metabolic pathways (Table 2 and Fig. 2A).

**Table 2:** List of top 50 genes downregulated and upregulated by *Notch1* haploinsufficiency. Fold change: up >2 fold. Fold change: down <0.5 fold.

S. No.	Gene ID	Gene name	Notch1^+/−^ Mφ vs WT-Mφ
1	15109	*Hal*	0.06
2	16008	*Igfbp2*	0.07
3	66857	*Plbd1*	0.09
4	219148	*Fam167a*	0.14
5	19752	*Rnase1*	0.17
6	12029	*Bcl6b*	0.18
7	27528	*Nrep*	0.18
8	19848	*Rnu2–10*	0.19
9	107526	*Gimap4*	0.19
10	170574	*Sp7*	0.20
11	21838	*Thy1*	0.21
12	66695	*Aspn*	0.21
13	230766	*Fam167b*	0.22
14	74144	*Robo4*	0.23
15	17285	*Meox1*	0.23
16	12097	*Bglap2*	0.24
17	84094	*Plvap*	0.24
18	12338	*Capn6*	0.24
19	12363	*Casp4*	0.24
20	11647	*Alpl*	0.24
21	11522	*Adh1*	0.25
22	12096	*Bglap*	0.25
23	13120	*Cyp4b1*	0.25
24	114249	*Npnt*	0.25
25	13876	*Erg*	0.26
26	19223	*Ptgis*	0.26
27	76829	*Dok5*	0.27
28	333883	*Cd59b*	0.27
29	15891	*Ibsp*	0.27
30	246316	*Lgi2*	0.27
31	12741	*Cldn5*	0.27
32	257635	*Sdsl*	0.27
33	69524	*Esam*	0.28
34	54003	*Nell2*	0.28
35	231655	*Oasl1*	0.28
36	13396	*Dlx6*	0.28
37	59308	*Emcn*	0.28
38	320415	*Gchfr*	0.28
39	20672	*Sox18*	0.28
40	21846	*Tie1*	0.28
41	18654	*Pgf*	0.29
42	14264	*Fmod*	0.29
43	243277	*Adgrd1*	0.29
44	64074	*Smoc2*	0.29
45	16011	*Igfbp5*	0.29
46	12904	*Crabp2*	0.29
47	24110	*Usp18*	0.30
48	27047	*Omd*	0.31
49	268958	*Capn11*	0.31
50	56198	*Heyl*	0.31
1	27209	*Snord32a*	8.15
2	232714	*Mgam*	7.08
3	54725	*Cadm1*	5.67
4	27210	*Snord34*	5.33
5	19871	*Rnu73b*	5.07
6	18128	*Notch1*	4.90
7	19225	*Ptgs2*	4.43
8	387224	*Mir29c*	4.15
9	19874	*Rny3*	4.00
10	723963	*Mir29b-2*	3.52
11	436188	*Gm5751*	3.50
12	71988	*Esco2*	3.25
13	208628	*Kntc1*	3.20
14	170947	*Myoz3*	3.19
15	16323	*Inhba*	2.83
16	67121	*Mastl*	2.82
17	67629	*Spc24*	2.77
18	64337	*Gng13*	2.70
19	387216	*Mir23a*	2.70
20	270120	*Fat3*	2.68
21	11609	*Agtr2*	2.62
22	432855	*Zfhx2os*	2.62
23	16551	*Kif11*	2.55
24	12771	*Ccr3*	2.51
25	70218	*Kif18b*	2.51
26	387164	*Mir146*	2.45
27	244218	*Ctf2*	2.42
28	103142	*Rdh9*	2.42
29	208292	*Zfp871*	2.41
30	212391	*Lcor*	2.33
31	218581	*Depdc1b*	2.31
32	108912	*Cdca2*	2.31
33	547347	*Gm6034*	2.30
34	17345	*Mki67*	2.30
35	97908	*Hist1h3g*	2.28
36	72415	*Sgol1*	2.27
37	77777	*Ulbp1*	2.26
38	210530	*P3h2*	2.25
39	20311	*Cxcl5*	2.24
40	78316	*Platr27*	2.22
41	387235	*Mir125a*	2.21
42	449630	*Snord15a*	2.21
43	15258	*Hipk2*	2.21
44	70466	*Ckap2l*	2.20
45	270160	*Rab39*	2.19
46	54615	*Npff*	2.19
47	240641	*Kif20b*	2.16
48	654810	*Appbp2os*	2.16
49	218977	*Dlgap5*	2.16
50	387158	*Mir140*	2.16

*Notch1* haploinsufficiency also upregulated the baseline expression of ~100 genes by two-fold or more in the absence of external stimulants (Table 2; right red panel and data not shown). The genes relevant to macrophage polarization in this category included maltase-glucoamylase (*Mgam*), guanine nucleotide binding protein 13 (*Gng13*), and γ-parvin (*Parv-γ*). The important pathways affected by the upregulation of these genes included cell cycle, chemokine signaling pathway and cancer (Fig. 2A and data not shown).

### Notch1 haploinsufficiency dysregulates selective genes in response to LPS/IFN-γ treatment

Wide-ranging effects of LPS/IFN-γ were observed in ~2000 genes with a cutoff of a two-fold increase. The top 100 genes robustly upregulated in response to LPS/IFN-γ treatment ranged from hundreds to thousands fold relative to naïve Mφ (Supplemental Table 1). Upregulation of M1-genes including, chemokine (C-X-C motif) ligand 9 (*Cxcl9*), nitric oxide synthase 2, inducible (*Nos2*), immunoresponsive gene 1 (*Irg1*), *Cxcl1*0, interleukin 6 (*Il6*), *Il12b*, formyl peptide receptor 2 (*Fpr2*), tumor necrosis factor (*Tnf*), *Fpr1* and *Has1* was observed in WT BMDMs following LPS/IFN-γ treatment relative to baseline levels. Comparable increase in the expression of these genes was also observed in *Notch1*^+/−^ BMDMs with LPS/IFN-γ treatment but the extent of the increase was attenuated significantly in some genes including *Cxcl9*, *Irg1*, *Cxcl10*, *Fpr2* and *Has1 (P* < *0*.*01)*. Interestingly, we observed 50% or more reduction in the expression of ~100 genes with *Notch1* haploinsufficiency in BMDMs treated with LPS/IFN-γ compared to WT-BMDMs treated with LPS/IFN-γ (Table 3; left green boxes). This category included colony stimulating factor 2 (*Csf2*), lymphocyte antigen 6 complex, locus G (*Ly6g*), interleukin 1 receptor-like 1 (*Il1r1*), interleukin 2 receptor, beta chain (*Il2rb*), *Has1*, *Casp*4, lipopolysaccharide binding protein (*Lbp)* and *Ccl19* genes which have potential associations with macrophage polarization.

**Table 3:** List of top genes dysregulated by Notch1 haploinsufficiency in the presence of LPS/IFN-γ and IL4.

Gene Name	WT LPS/IFN-γ vs WT-Mφ	Notch1^+/−^ LPS/IFN-γ vs Notch1^+/−^ Mφ	Notch1^+/−^ Mφ vs WT-Mφ	Notch1^+/−^ LPS/IFN-γ vs WT- LPS/IFN-γ
*Snord14c*	5.66	0.23	2.02	0.08
*Timd4*	12.32	1.16	1.00	0.09
*Mir214*	2.43	0.31	0.82	0.11
*Rny3*	2.72	0.08	4.00	0.12
*Snord32a*	6.93	0.12	8.15	0.14
*Snhg9*	2.07	0.28	1.54	0.21
*Rnu1a1*	2.37	0.94	0.56	0.22
*Gimap9*	6.68	1.46	1.02	0.22
*Bdkrb1*	6.87	2.99	0.63	0.27
*Csf2*	9.77	2.73	1.00	0.28
*Snord15a*	2.26	0.29	2.21	0.28
*Rnu1b2*	3.54	1.00	1.00	0.28
*Bco2*	2.35	1.39	0.48	0.28
*Ankrd1*	2.79	0.83	0.97	0.29
*Rny1*	5.54	1.70	0.95	0.29
*Meikin*	16.35	4.80	1.00	0.29
*Lrrc73*	4.22	0.97	1.31	0.30
*Hist1h3a*	2.07	0.46	1.37	0.30
*Gabrd*	3.17	1.04	0.93	0.31
*Slc25a18*	5.69	1.51	1.16	0.31
*Ly6g*	3.17	1.00	1.00	0.32
*Il1rl1*	7.75	2.67	0.95	0.33
*Scin*	4.87	1.60	1.00	0.33
*Il2rb*	3.00	1.28	0.80	0.34
*Crnde*	2.23	1.03	0.74	0.34
*Hba-a2*	4.55	1.39	1.14	0.35
*Gzmc*	2.22	0.40	1.94	0.35
*Slc36a2*	7.64	3.02	0.89	0.35
*Has1*	29.65	22.78	0.46	0.35
*Mir101a*	2.60	2.05	0.46	0.36
*Tyro3*	2.12	1.23	0.63	0.37
*Calb2*	201.21	74.30	1.00	0.37
*Hba-a1*	3.80	1.41	1.00	0.37
*Lbp*	13.65	4.41	1.16	0.37
*Gimap4*	5.63	11.21	0.19	0.38
*Madcam1*	20.43	8.79	0.88	0.38
*Casp4*	15.46	24.54	0.24	0.38
*Tmem132e*	26.36	10.15	1.00	0.38
*Dact2*	4.84	2.60	0.72	0.39
*Cdhr1*	2.26	0.92	0.95	0.39
*Fam84a*	4.90	1.92	1.00	0.39
*Hs3st1*	3.86	2.27	0.68	0.40
*Spink2*	14.66	5.90	1.00	0.40
*Tnfrsf18*	2.74	1.48	0.74	0.40
*Mmp8*	0.10	0.18	1.94	3.25
*Mmp12*	0.12	0.18	1.55	2.21
*Tlr8*	0.06	0.08	1.64	2.18
*Lipn*	0.17	0.18	2.03	2.15
*Mrc1*	0.06	0.08	1.55	2.06
*Dhrs9*	0.30	0.37	1.63	2.04
**Gene Name**	**WT IL4/IL13 vs WT-Mφ**	**Notch1** ^**+/−**^ **IL4/IL13 vs** **Notch1** ^**+/−**^ **Mφ**	**Notch1** ^**+/−**^ **Mφ vs** **WT-Mφ**	**Notch1** ^**+/−**^ **IL4/IL13 vs** **WT IL4/IL13**
*Prl2c3*	2.31	9.00	1.13	4.40
*Prl2c2*	3.16	14.48	0.93	4.26
*Snord32a*	3.01	1.09	8.15	2.94
*Snora74a*	2.44	4.71	1.40	2.70
*Oc-stamp*	4.14	10.82	1.00	2.61
*Hist1h3g*	2.34	2.13	2.28	2.07
*Car3*	2.27	4.93	0.94	2.04
*Hal*	0.36	0.59	0.06	0.10
*Sox18*	0.49	0.45	0.28	0.26
*Dio3*	0.28	0.08	1.02	0.28
*Slc24a3*	0.32	0.12	0.84	0.32
*Itgbl1*	0.34	0.16	0.76	0.37
*Rnase1*	0.49	1.12	0.17	0.38
*Bcl6b*	0.41	0.90	0.18	0.39
*Kcnmb4os1*	0.42	0.43	0.39	0.39
*Tgfbi*	0.43	0.15	1.25	0.43
*Robo4*	0.44	0.83	0.23	0.43
*Cyp4b1*	0.31	0.56	0.25	0.45
*Nid2*	0.37	0.21	0.77	0.45
*Thsd7a*	0.46	0.27	0.78	0.46
*Plxna4os1*	0.37	0.28	0.61	0.46
*Oas2*	0.49	0.46	0.49	0.47
*Sdsl*	0.39	0.66	0.27	0.47
*Egfl7*	0.43	0.53	0.37	0.47
*Emcn*	0.44	0.74	0.28	0.47
*Cldn1*	0.43	0.21	0.96	0.48
*Rasip1*	0.46	0.57	0.39	0.48
*Spata33*	0.40	0.14	1.33	0.48
*Gpr31b*	0.41	0.37	0.52	0.48
*Tmem121*	0.43	0.33	0.66	0.50

In response to LPS/IFN-γ treatment, downregulation of ~4000 genes below 50% of the baseline expression was observed in *WT* and *Notch1*^+/−^ BMDMs (Supplemental Table 1). The important genes/families in this category were regulator of G-protein signaling 18 (*Rgs18*), brain expressed X-linked 1 and 4 (*Bex1* and *Bex4*), *CD28*, peroxisome proliferator activated receptor gamma (*Ppar-γ*), *Il4*, SRY-box 9 (*Sox9*), transforming growth factor beta 2 (*Tgf-β2*), *Mmp8*, *Mmp12*, Tgf-β receptor II (*Tgfβ-RII*) and *Tgfβ-RI*. Out of these 4000 genes, downregulated by LPS/IFN-γ, about 25 genes were significantly upregulated by *Notch1* haploinsufficiency (Table 3). Noticeable genes in this category include mannose receptor C1 (*Mrc1*), dehydrogenase/reductase member 9 (*Dhrs9*), *Mmp8*, *Mmp12*, *Tlr8* and *Lipn*. The alteration in the expression of these genes with *Notch1* haploinsufficiency suggests possible roles of *Notch1* signaling in the macrophage polarization through these novel genes.

### Notch1 haploinsufficiency dysregulates selective genes in response to IL4/IL-13 treatment

IL4/IL13 induced expression of distinct set of genes, which are known to play a significant role in the resolution of inflammation (Supplemental Table 2). This category included chitinase-like (*Chil3*,*4*,5,6), retinol binding protein 4 (*Rbp4*), IL13 receptor, alpha 2 (*Il13ra2*), *Irf4*, *Arg1*, suppressor of cytokine signaling 1 (*Socs1*), macrophage galactose N-acetyl-galactosamine specific lectin 2 (*Mgl2*) and *Ccl12* genes. *Notch1* haploinsufficiency also increased the expression of these genes in response to IL4/IL13 and the magnitude of upregulation was even higher. *Notch1* haploinsufficiency also increased the expression of prolactin family members c2 and c3 (*Prl2c2* and *Prl2c3*), small nucleolar RNA C/D box 32 A (*Snord32a*), small nucleolar RNA H/ACA box 74 A (*Snora74a*), osteoclast stimulatory transmembrane protein (*Oc-stamp*) and carbonic anhydrase 3 (*Car3*) significantly in response to IL4/IL13 treatment compared to WT BMDMs with similar regimen (Table 3).

IL4/IL13 treatment downregulated expression of ~800 genes to less than 50% of baseline expression in both *WT* and *Notch1*^+/−^ mice (Supplemental Table 2). The genes in this category which may have potential roles in macrophage polarization were aldo-keto reductase family 1 (*Akr1c18*), Mmp3, Fgr proto-oncogene (*Fgr*), bone morphogenetic protein 6 (*Bmp6*), *Mmp1*0, allograft inflammatory factor 1 (*Aif*), angiotensin I converting enzyme (*Ace*), early growth response 3 (*Egr3*), and intercellular adhesion molecule 1 (*Icam1*). With *Notch1* haploinsufficiency, there was further downregulation of histidine ammonia lyase (*Hal*), Sox18, plexin A4 opposite strand 1 (*Plxna4os1*), *Oas2* and *Gpr31b* to less 50% of the WT BMDMs with IL4/IL13 treatment (Table 3). These data suggest that *Notch1* haploinsufficiency may be affecting macrophage polarization through these novel genes (Fig. 2C).

### Notch1 haploinsufficiency downregulates unique genes; differential gene expression analysis and RT-qPCR validation

**Figure 3:**
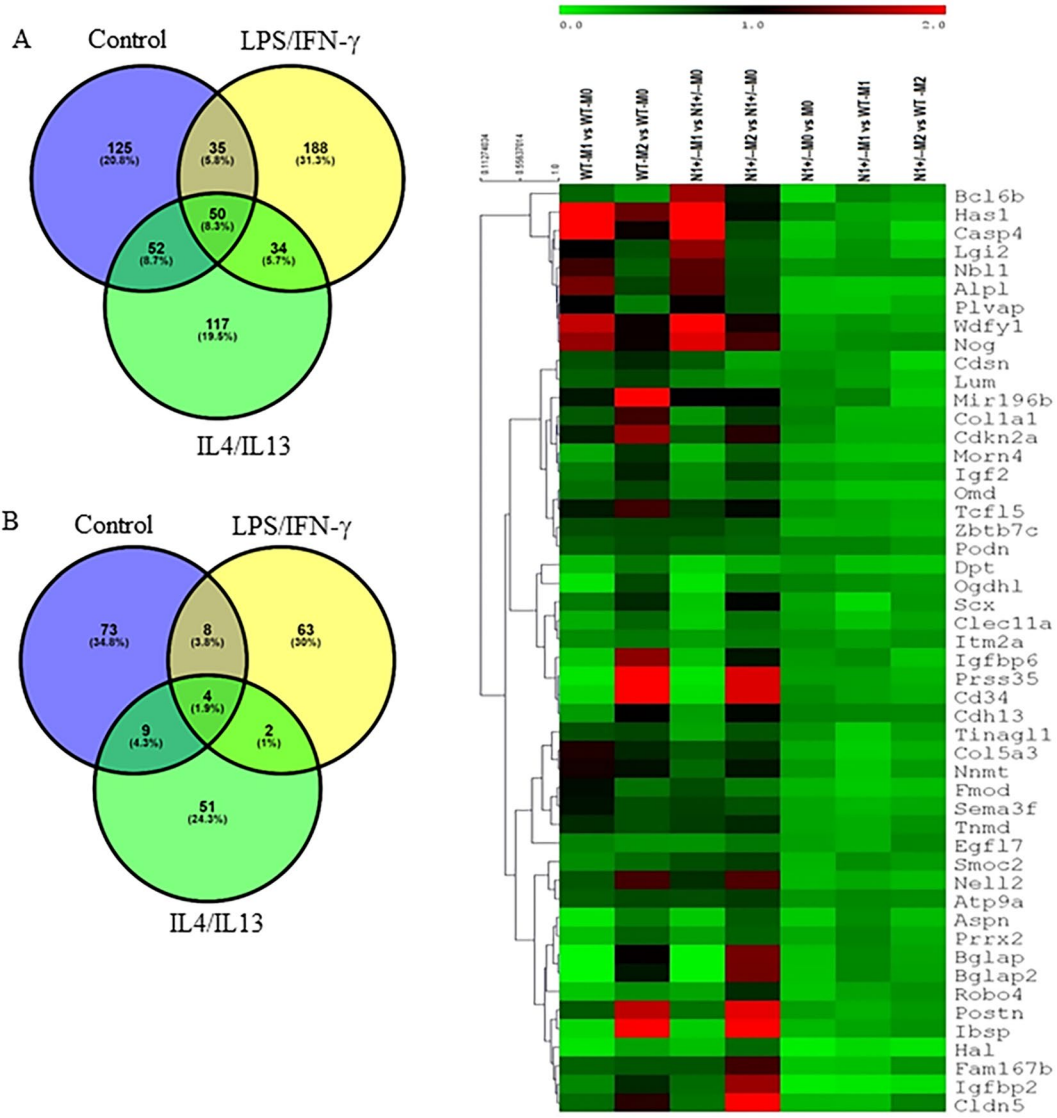
Differentially expressed genes (DEGs) analysis revealed a set of unique genes dysregulated by *Notch1* haploinsufficiency. (**A**) Venn diagram showing the number of genes downregulated (less than 0.5-fold) in *Notch1*^+/−^ BMDMs treated with vehicle, LPS-IFN-γ or IL4/IL13 to WT BMDMs with similar regimen. (**B**) Venn diagram showing the number of genes upregulated (more than 2-fold) in *Notch1*^+/−^ BMDMs treated with vehicle, LPS/IFN-γ or IL4/IL13 to WT with similar regimen. Each portion of a Venn diagram displays number of DEGs in *Notch1*^+/−^ BMDMs compared to *WT* BMDMs. (C) Heat map from 50 common mouse genes downregulated in *Notch1*^+/−^ BMDMs with all the treatments than WT BMDMs with similar regimen.

**Figure 4:**
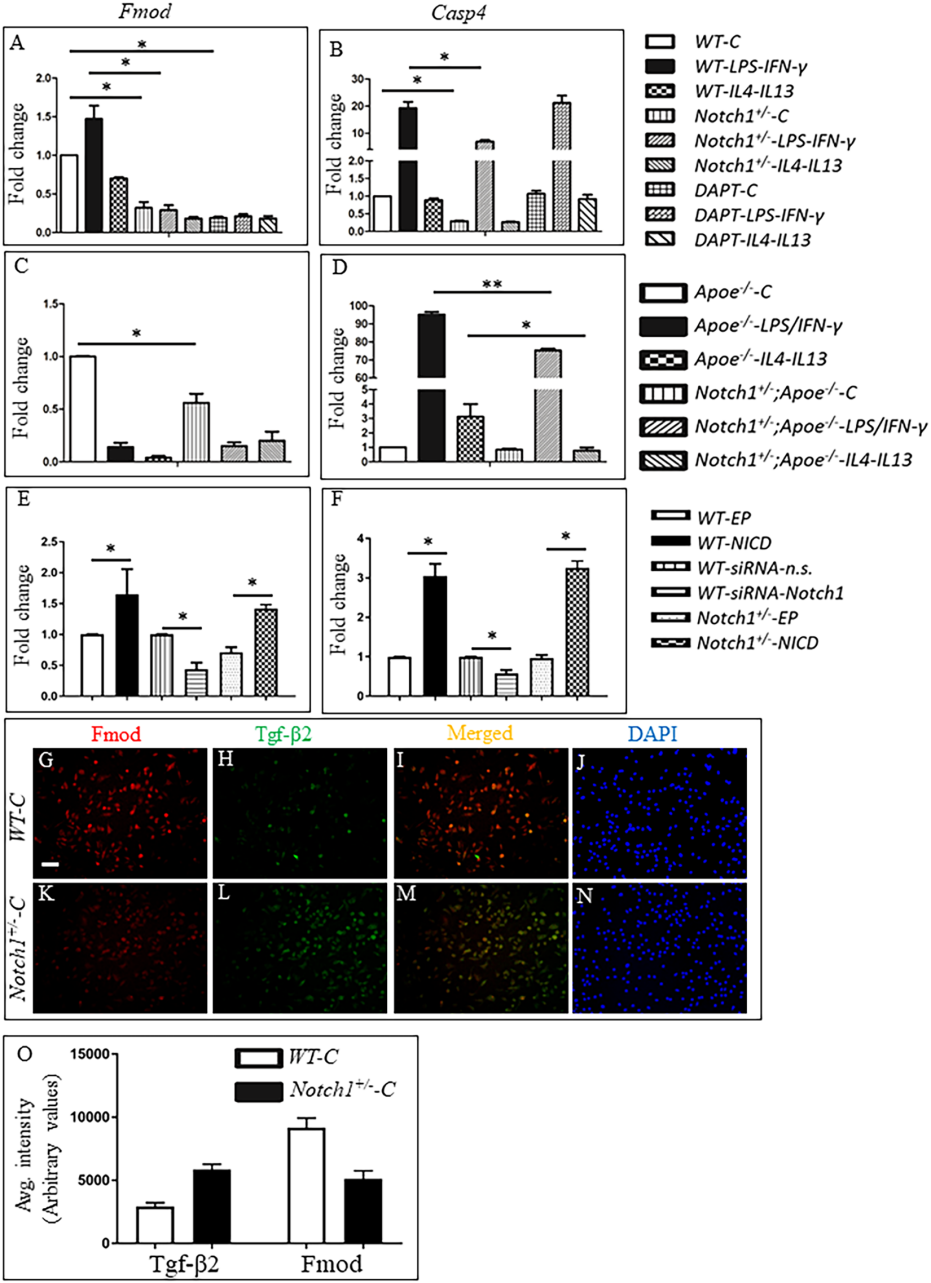
Quantitative real-time PCR validates the RNA-Seq data for fibromodulin (*Fmod*) and Caspase-4 (*Casp4*). (**A**,**B**) Bar graphs represent fold change in gene expression of *Fmod* and *Casp4* in WT, *Notch1*^+/−^ BMDMs and WT + DAPT (a potent inhibitor of Notch signaling). BMDMs were pre-treated with DMSO or DAPT for 24 h followed by LPS/IFN-γ or IL4/IL13 for 24 h. (**C**,**D**) Bar graphs represent the gene expression of *Fmod* and *Casp4* in *Apoe*^−/−^, *Notch1*^+/−^*;Apoe*^−/−^ BMDMs treated with LPS/IFN-γ or IL4/IL13 for 24 h. (**E**,**F**) Graphs represent fold change in the expression of *Fmod* and *Casp4* in WT, or *Notch1*^+/−^ BMDMs 48 h post transfection with Notch1 intracellular domain (NICD) plasmid or siRNA-Notch1. Gene expression was determined using qPCR, normalized to *Rpl13a* and reported as fold change (mean ± SEM, n = 3 for each group) to WT-C or *Apoe*^−/−^C. (**G**–**N**) Double immunofluorescence (DIF) staining of BMDMs revealing the pattern of expression of Fmod (**G**,**K**), Tgf-β2 (**H**,**L**) and their merged images (**I**,**M**). Nuclei are shown in blue DAPI staining (J, N). (O) Quantification of DIF staining of BMDMs using Lionheart FX Gene5 software. The data is represented as average intensity of ~200 cells from each group. Original magnification 40 × , Scale bars = 50 µm. ***p < *0*.001, **p < 0.01, *p < 0.05 (one way ANOVA followed by a Tukey’s multiple comparisons test comparisons test). (EP = empty plasmid).

Differentially expressed genes (DEGs) analysis revealed a set of unique genes, which were dysregulated by *Notch1* haploinsufficiency at baseline or with different treatments (Table 1). Uninformative genes were removed from initial filtering to minimize the computational graphics and to obtain distinct groups of genes for subsequent analysis. *Notch1* haploinsufficiency downregulated the expression of 262 genes under baseline conditions, 307 genes with LPS/IFN-γ and 254 genes with IL4/IL13 treatment (Fig. 3A). *Notch1* haploinsufficiency upregulated the expression of 94 genes at baseline conditions, 77 genes with LPS/IFN-γ and 66 genes with IL4/IL13 treatment (Fig. 3B). Venn diagram analysis of these DEGs revealed 50 unique genes that were significantly downregulated by *Notch1* haploinsufficiency (Fig. 3A,C and Table 1). Functional enrichment analysis and hierarchal clustering predicted that DEGs were associated with a number of pathways involved in macrophage polarization (*Casp4*, *Has1*, *Cd34*, *Cdh5*, *Fmod*, *Lum*, *Nbl1*, *Postn*, *Plvap*), ECM degradation (*Col1a1*, *col5a3*, *Fmod*, *Lum*) and osteogenesis (*Alpl*, *Igf2*, *Igfbp2*) (Figs 2C, 3C). The gene expression of *Fmod*, *Casp4*, *Alpl*, *Col1a1*, *Igf-bp2* and *Lum* were validated by real-time qRT-PCR and the data were consistent with the RNA-Seq analyses (Fig. 4A,B and data not shown).

With regard to the expression of Notch1, the qRT-PCR and RNA-Seq data revealed contrasting results. The decreased expression of Notch1 was confirmed in the *Notch1*^+/−^ macrophages by genotyping and qRT-PCR (Supplemental Fig. 3C–F). The downstream target of Notch1 *HeyL* and its ligand *Dll4* were significantly downregulated in both the RNA-Seq and qRT-PCR data confirming that the changes reflected in the RNA-Seq data can be attributed to reduced Notch1 signaling (Supplemental Fig. 1C and Table 2). Moreover, reduction in the downstream targets of Notch1 signaling was also observed in response to DAPT and Notch1 siRNA albeit at different extent (Supplemental Fig. 3E,F). Overexpression of Notch1 by NICD plasmid on the other hand increased the expression of HeyL, Hes1, Hey1 and Hey2 and Jag1 significantly in the unstimulated macrophages (Supplemental Fig. 3D). These changes were also reflected in the total protein contents of NICD (Supplemental Fig. 3G). Interestingly, RNA-Seq data revealed that the expression of Notch1 was higher in the *Notch1*^+/−^ macrophages than the WT BMDMs. One potential explanation of this discrepancy could be the instability of Notch1 mRNA and susceptibility to degrade in different conditions^35–37^. The evidence for these conflicting observations was demonstrated by the number of reads in WT samples which were strong at the 3′ region (the polyA tail region), but diminished towards the 5′ end of the transcripts (Supplemental Fig. 4A). As the mRNA was enriched by polyA binding during library preparation, only mRNAs with an intact polyA tail would have been included in our sequencing library. Moreover, treatment of macrophages with Actinomycin D and HuR significantly increased expression of Notch1, whereas no significant effect of these stimulants on *Dll4* or *Fmod* was observed (Supplemental Fig. 4B). The analysis of Ensembl Exon numbers for the full-length coding Notch 1 transcript indicate a logical divergence in the results of the *Notch1* expression as determined by qRT-PCR or RNA-Seq analysis (Supplemental Fig. 3C–G). Increased read-counts for Notch 1 mRNA expression were only observed in exons 1–19 of the *Notch1*^+/−^ BMDMs, the undeleted portion of Notch1 allele^38^. Starting with exon 20–33, very little expression of Notch1 was observed, which is consistent with the generation of mutated *Notch1*^+/−^ mouse model^38^. Although, these observations are very intriguing, further studies are required to determine the role of Notch1 signaling on RNA integrity using epigenetic and functional approaches. In the following experiments, we focused on the unique genes that were downregulated by *Notch1* haploinsufficiency.

### Gain and loss-of-function studies suggest that Notch1 directly regulates Fmod and Casp4

Among the 50 genes downregulated by Notch1 haploinsufficiency, *Fmod* and *Casp4* were further evaluated because of their potential involvement in macrophage polarization^39–41^. In agreement with the RNA-Seq results, downregulation of expression for *Fmod* (Fig. 4A) and *Casp4* (Fig. 4B) *was* confirmed in the BMDMs from *Notch1*^+/−^ mice by real-time qRT-PCR. DAPT, a potent inhibitor of Notch signaling also reduced the gene expression of Fmod to less than 50% of the baseline levels (Fig. 4A). However, expression of *Casp4* was not significantly altered with DAPT treatment (Fig. 4B). Decreased expression *Fmod* and *Casp4* genes was also observed in *Apoe*^−/−^ BMDMs by *Notch1* haploinsufficiency suggesting that these effects were global and independent of the strain (Fig. 4C,D). Next, we performed gain and loss-of-function studies using NICD plasmids or specific Notch1 siRNA respectively (Fig. 4E,F). Overexpression of Notch1 by NICD plasmid increased the expression of *Fmod* by almost 1.5 fold, whereas Notch1 siRNA reduced the expression of *Fmod* below 50% (Fig. 4E,F). Co-immunostaining with Fmod and Tgf-β2 demonstrated strong immunoreactivity for Fmod in WT BMDMs with a concomitant weak immunoreactivity for Tgf-β2 (Fig. 4G–N). On the contrary, in *Notch1*^+/−^ BMDMs, weak immunoreactivity for Fmod and strong immunoreactivity for Tgf-β2 was observed (Fig. 4K,N). The quantitation of double immunofluorescence (DIF) showed inverse correlation between Tgf-β2 and Fmod immunoreactivity in WT and *Notch1*^+/−^ BMDMs (Fig. 4O). Collectively, our data clearly suggest that Notch1 is involved in the modulation of Fmod expression.

**Figure 5:**
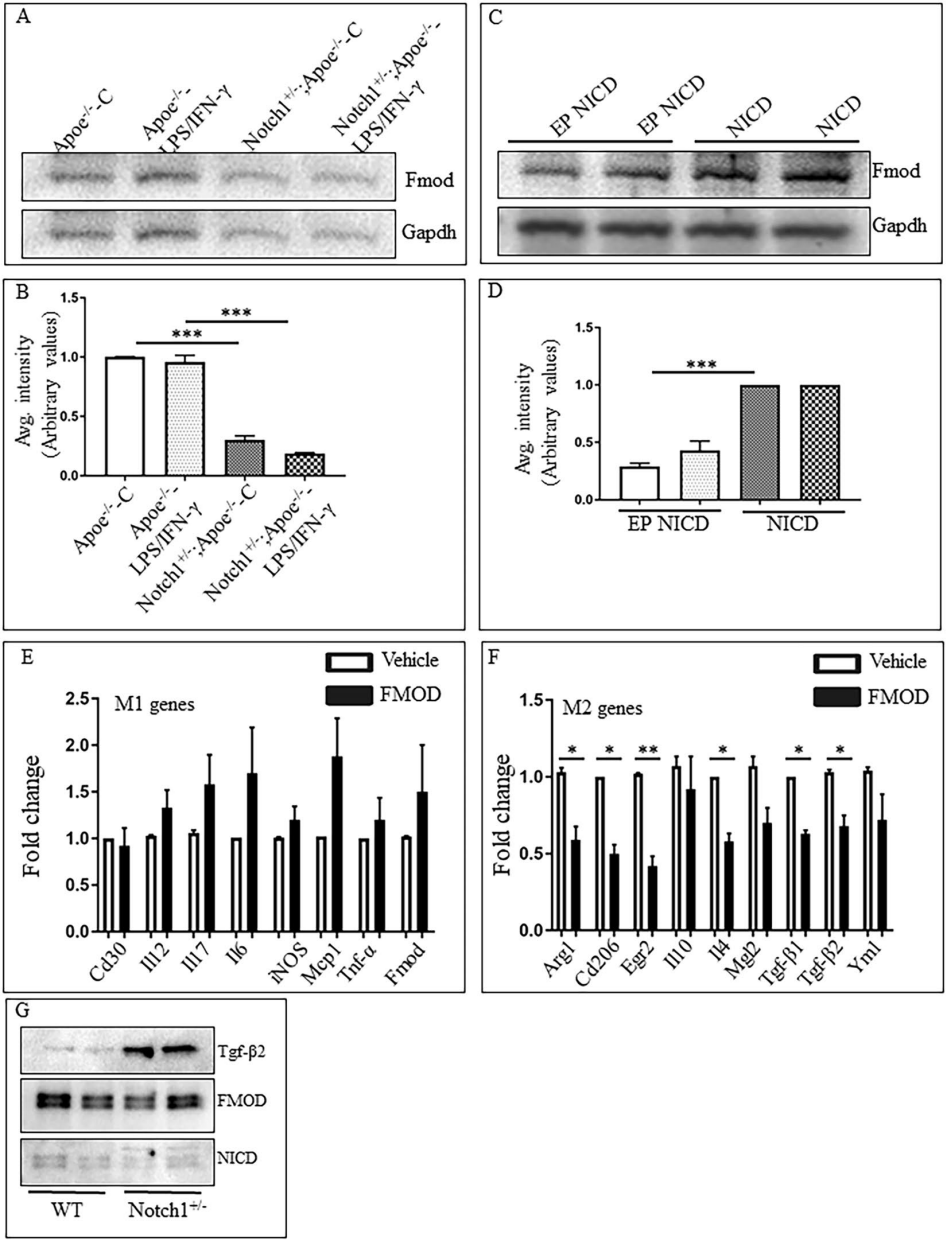
Overexpression of NICD increases the total Fmod protein contents in BMDMs and LPS prevents the cleavage of Fmod in a dose dependent manner. (**A**,**B**) WB shows the expression of total Fmod in *Apoe*^−/−^ and *Notch1*^+/−^*;Apoe*^−/−^ BMDMs and quantification of three replicates as determined by Image J. (**C**,**D**) WB showing the total Fmod protein content in *WT* BMDMs 48 h post transfection with empty or NICD plasmids and the quantification of the immunoblots. (**E**,**F**) qRT-PCR showing the panel of M1 and M2 genes dysregulated with human recombinant FMOD (400 ng/ml for 24 h). (**G**) Co-immunoprecipitation showing contents of Tgf-β2, Fmod and NICD proteins pulled down with Fmod antibody from the *WT* and *Notch1*^+/−^ peritoneal macrophages.

Further, we confirmed that *Notch1* haploinsufficiency markedly decreased the Fmod protein content significantly, both at baseline levels and in response to LPS/IFN-γ treatment (Fig. 5A,B). Overexpression of NICD in these BMDMs significantly increased the Fmod protein contents (Fig. 5C,D). Treatment of macrophages with human recombinant FMOD (400 ng/ml; 24 h) significantly decreased the expression of common M2 genes including *Tgf-β1*, *Tgf-β2*, *Arg1*, *Cd206* and *Il4* (Fig. 5F). Interestingly, no significant effect of FMOD on M1 genes was observed in these settings (Fig. 5E), suggesting that these effects of FMOD on M2-polarization may be mediated by Tgf-β2. To confirm direct interactions between FMOD and Tgf-β2, we performed immunoprecipitation studies using Dynabeads^®^ Co-Immunoprecipitation Kit (ThermoFisher). Western blotting of the immunoprecipitate with FMOD revealed the presence of Tgf-β2 in the *Notch1*^+/−^ macrophages, whereas in the WT macrophages, the contents of Tgf-β2 were minimal (Fig. 5G). These data confirm that FMOD protein is physically associated with Tgf-β2 and also provide evidence for increased contents of Tgf-β2 in *Notch1* haploinsufficient macrophages.

**Figure 6:**
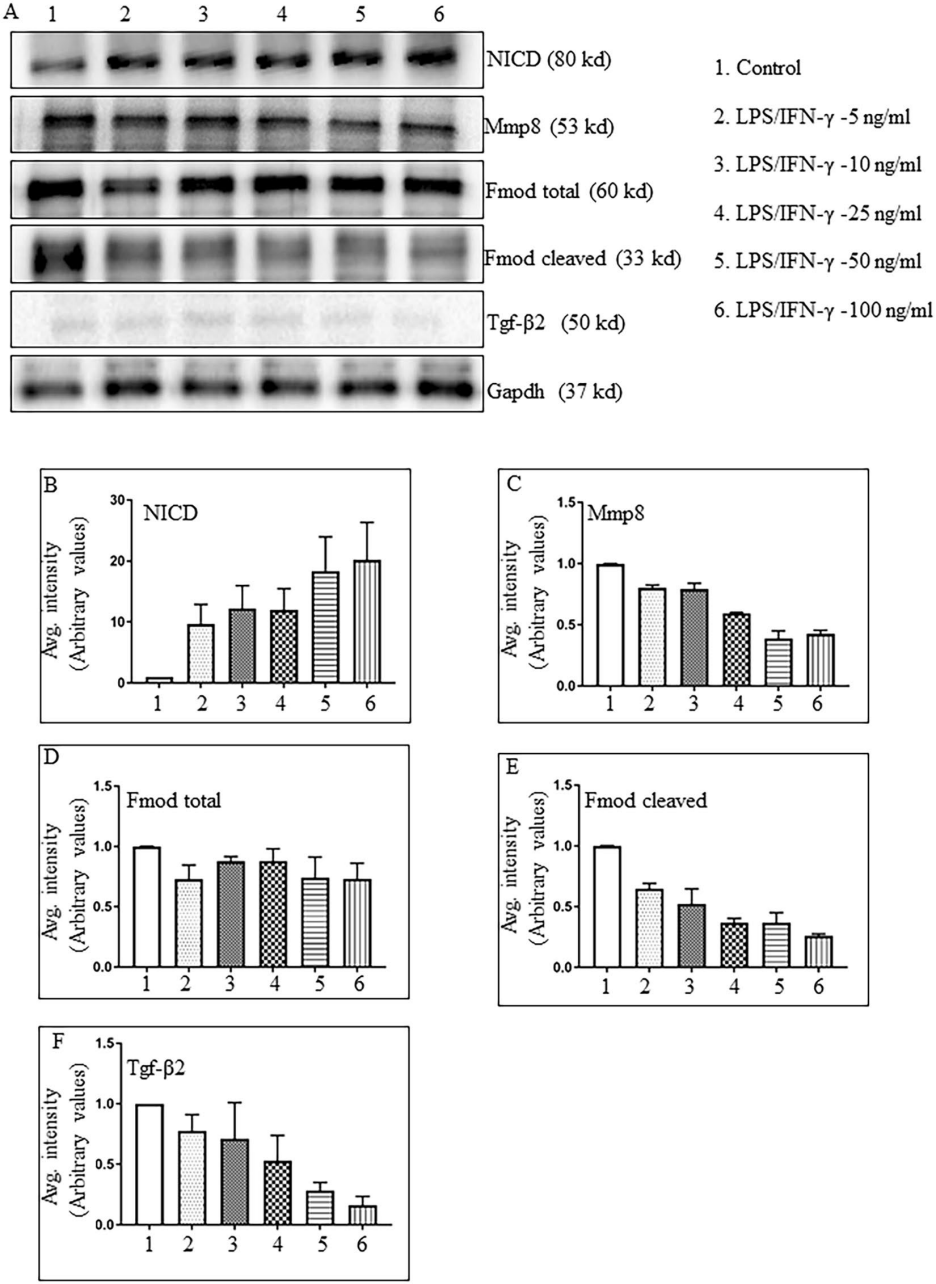
Dose-dependent effects of LPS/IFN-γ on Tgf-β expression are associated with changes in the cleaved Fmod fragments. (**A**) Representative WB image showing the contents of NICD, Mmp8, total Fmod, cleaved Fmod, Tgf-β2 and Gapdh in macrophages in response to increasing dose of LPS (0, 5, 10, 25, 50 or 100 ng/ml) for 24 h. (**B**–**F**) Quantitation of immunoblots for NICD, Mmp8, total Fmod, cleaved Fmod, Tgf-β2 respectively (average of three replicates shown after normalizing the intensity with Gapdh. (WB = Western blot).

As demonstrated by qRT-PCR (Fig. 4A–D) and Western blot (Fig. 5A–D and Supplemental Fig. 5A), the expression of Fmod was not significantly affected by LPS/IFN-γ treatment. LPS/IFN-γ treatment reduces Tgf-β signaling as demonstrated by various studies, but the mechanism is unknown^20,21,42,43^. We determined if these effects of LPS/IFN-γ on Tgf-β are mediated by Fmod. Macrophages were exposed to differing concentrations of LPS (0, 5, 10, 25, 50 or 100 ng/ml) and 20 ng/ml of IFN-γ for 24 h, thereafter protein extracts were examined by Western blotting. LPS treatment did not change total Fmod protein content (60 kd), but a dose-dependent decrease in the cleaved Fmod fragment was observed (33kd; Fig. 6A–F and Supplemental Fig. 5B). A concomitant decrease in the Tgf-β2 protein content was also observed with LPS/IFN-γ treatment (Fig. 6A,F). Proteolysis of Fmod through Mmp8 has been shown to increase the expression of Tgf-β2^39^. We also observed a dose-dependent decrease in the Mmp8 content, which correlated directly with Tgf-β2 expression and inversely with NICD expression (Fig. 6A,C).

**Figure 7:**
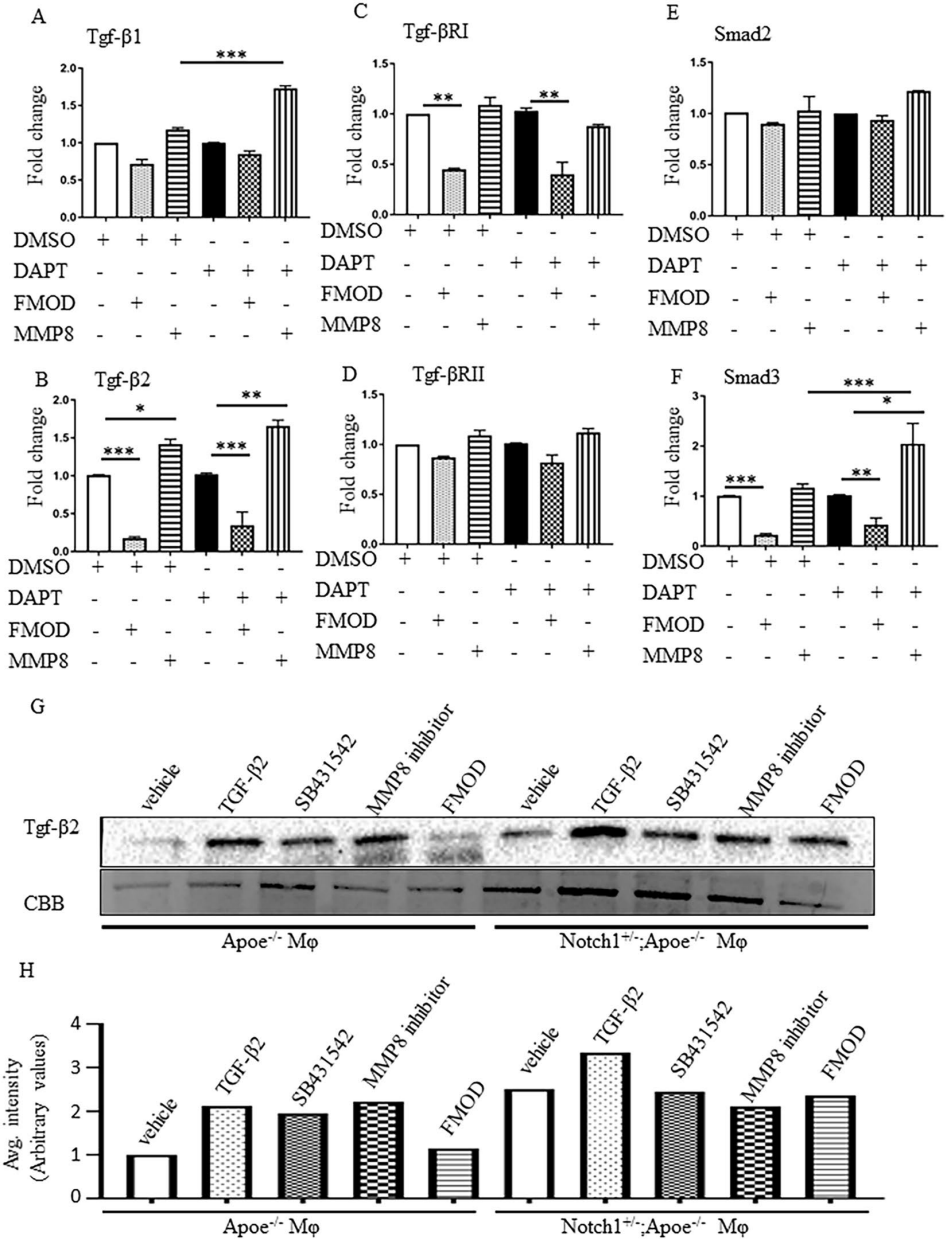
FMOD decreases the expression of Tgf-β2 signaling pathway in BMDMs. Gene expression of the members of Tgf-β signaling pathway-Tgf-β1 (**A**), Tgf-β2 (**B**), Tgf-βRI (**C**), Tgf-βRII (**D**), Smad2 (**E**) and Smad3 (**F**) was determined using real-time PCR in WT-BMDMs pre-treated with DMSO or DAPT (10 µM) for 24 h followed by treatment with FMOD (400 ng/ml) or activated-MMP8 (500 ng/ml) for 24 h. Gene expression was determined using qPCR, normalized to *Rpl13a* and reported as ratio (mean ± SEM, n = 3 for each group) to DMSO-C or DAPT-C. (**G**,**H**) WB of the media concentrated from *Apoe*^−/−^ and *Notch1*^+/−^*;Apoe*^−/−^ BMDMs treated with TGF-β2 (human recombinant protein; 5 ng/ml), SB431542 (an inhibitor of activin receptor-like kinase; 15 nM), MMP8 inhibitor (10 nM) or FMOD (100 ng/ml) for 24 h and the quantification. The media was concentrated 50 fold and 20 µl volume was loaded for the WB for secreted Tgf-β2. Coomassie Brilliant Blue G-250 Dye (CBB) was used to demonstrate equal loading of the media. ***P < 0.001, **p < 0.01, *p < 0.05 (one way ANOVA followed by a Tukey’s multiple comparisons test). (WB = Western blot).

Finally, we determined if Notch1 signaling affects the expression of downstream targets of the Tgf-β signaling pathway and if this was mediated by Fmod. BMDMs from WT or *Notch1*^+/−^ were treated with either FMOD (400 ng/ml) or activated MMP8 (400 ng/ml) for 24 h. Treatment with FMOD significantly decreased the expression of Tgf-β2 (0.18 ± 0.03; Fig. 7B), Tgf-βRI (0.45 ± 0.02; Fig. 7C) and Smad3 (0.23 ± 0.04; Fig. 7F), whereas treatment with activated-MMP8 significantly increased the expression of Tgf-β2 (1.42 ± 0.11; Fig. 7B) in macrophages. With DAPT pretreatment, there was lesser but insignificant decrease in expression of Tgf-β2 with FMOD treatment (0.35 fold; Fig. 7B). In response to MMP8, increased trend was observed in the Tgf-β2 expression but did not reach significance (1.66 ± 0.13; Fig. 7B). No change in the expression of Tgf-β1 or Smad3 was observed in DMSO-treated macrophages, whereas in DAPT-treated macrophages, the expression of Tgf-β1 (1.73 ± 0.06; Fig. 7A) and Smad3 (2.05 ± 0.70; Fig. 7F) increased significantly in response to MMP8. No significant changes in the expression of Tgf-βRI (Fig. 7C) or Smad2 (Fig. 7E) expression were observed in response to FMOD or MMP8 in DMSO or DAPT-treated macrophages suggesting that these contrasting effects of FMOD and MMP8 are selective and specific. We also determined the release of secreted Tgf-β2 in the media in response to various stimuli. FMOD did not affect the secreted Tgf-β2 protein in the media in *Apoe*^−/−^ BMDMs (Fig. 7G,H) whereas with *Notch1* haploinsufficiency, the secreted Tgf-β2 contents remained higher in the unstimulated as well as in the presence of FMOD (Fig. 7G,H).

## Discussion

**Figure 8:**
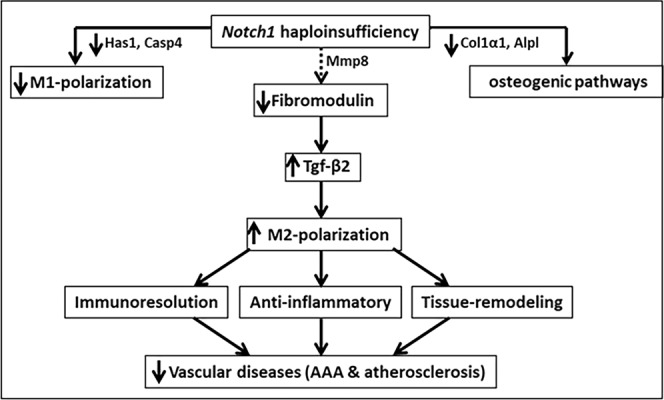
Schematic diagram of the study. The dashed arrows depict the mechanistic pathways suggested by the outcome of RNA-Seq and follow-up experiments in our study.

The concept of M1 and M2 polarization (classical and alternative, respectively), although very complex, is increasingly becoming relevant to the pathogenesis of a number of vascular and inflammatory diseases. The key factors that modulate polarization are not well understood, limiting the utilization of this concept as a therapeutic target. We have previously shown that haploinsufficiency of *Notch1* favors the transition of naïve (Mφ) macrophages to the anti-inflammatory M2-phenotype; however the mechanism is still obscure^19–21^. Using RNA-Seq and subsequent validation, we have identified 50 differentially expressed genes (DEGs) in *Notch1*^+/−^ macrophages under different treatments. Mapping these DEGs using the GO database^33^ and with KEGG pathways enrichment analysis^34^, we demonstrated that many of these novel genes are involved in biological pathways necessary during the initiation of inflammation, ECM pathway and macrophage polarization. Of particular note, gain and loss-of-function studies confirmed that Notch1 signaling regulates the expression of Fmod, a proteoglycan family protein, which is involved in Tgf-β mediated M2 polarization of macrophages. Fmod is a 42–80 kd cytosolic protein, belonging to the class 2 small leucine-rich proteoglycan family (SLRPs) and is involved in regulation of collagen fibrillogenesis, cell adhesion, modulation of cytokine activity, and prevention of apoptosis^44^. Recent reports suggest that Fmod binds to Tgf-β ligands, and a decrease in the total protein of Fmod, or increased degradation of Fmod by Mmp8 may cause increased activity of Tgf-β2^39,40,45^. However, the implications of such binding in macrophage polarization have not been elucidated. Because of close associations of Fmod with Tgf-β signaling pathways, we speculate that *Notch1* haploinsufficiency may be promoting M2 macrophages by regulating Fmod gene expression and/or its activity. In our studies, Fmod not only decreased the expression of Tgf-β2, but also decreased the expression of various genes associated with M2-polarization. Interestingly, no change in the expression of M1-related genes was observed in macrophages with the addition of Fmod suggesting that Fmod have specific roles in M2-poalrization of macrophages (Fig. 8). Further studies are required to confirm the direct crosstalk between Notch1 signaling and Fmod in the context of a disease model.

*Notch1* haploinsufficiency dysregulated numerous genes at baseline conditions or in response to stimulants. While the associations of some of these genes with Notch1 signaling have been described in the literature, our study revealed novel genes that may potentially play active roles in the M1 or M2 polarization of macrophages. Among the several genes downregulated by *Notch1* haploinsufficiency at baseline conditions, *Nog*, *Alpl* and *Col1a1* genes, seem to be the most prominent. These genes have been associated with osteogenic pathways, but their association with macrophage polarization is still emerging^46–48^. Among the other genes downregulated by *Notch1* haploinsufficiency, Has1 is critical for the synthesis of hyaluronan, a constituent of the vascular ECM and an important ligand for monocyte recruitment and retention^49^. Gimap4 belongs to GTPase of the immunity-associated protein family proteins which are most extensively expressed in the course of Th1 differentiation, and less so during Th2 differentiation. Among the genes upregulated by *Notch1* haploinsufficiency at baseline conditions, Mgam has recently been suggested to mediate alternative activation of macrophages in integrin-β6–deficient mice^50^. In addition, Gng13 belongs to a class of G protein–coupled receptors family and implicated in the activation of Tgf-β^51^. Thus, these findings are highly suggestive of the involvement of Notch1 signaling in macrophage functions through these novel genes.

*Notch1* haploinsufficiency reduced the expression of ~100 genes, which were upregulated with LPS/IFN-γ treatment in WT mice. While the literature has demonstrated relationships among *Timd4*, *Csf2*, *Ly6g*, *Il1r1*, *Madcam*, *Casp4* and *Ccl19* and macrophage polarization^52,53^, there is limited data regarding the association of these genes with Notch1 signaling. In addition, RNA-Seq data revealed new targets for *Notch1* haploinsufficiency on M1-polarization including *Has1*, *or Spink2*, *Cngb3* and *Kcna4*. The upregulation of several genes including Mrc1, Dhrs9, Mmp8, and Lipn by *Notch1* haploinsufficiency in the presence of LPS/IFN-γ suggest that Notch1 signaling may have wider applications in the polarization of macrophages. Mrc1 is a well-established modulator of alternatively activated M2 macrophages^54^. DHRS9 has very recently been identified as a specific and stable marker of human regulatory macrophage (Mregs)^55^. Mmp8, a relatively less studied member of the MMP family, has received increasing attention in recent years because of its involvement in M2-polarization by modulation Tgf-β bioavailability^39^. Macrophages derived Mmp8-deficient mice expressed higher levels of M1-polarization markers but lower levels of M2-polarization markers^39^. Taken together, these findings strengthen the causative roles of *Notch1* haploinsufficiency in the regulation of M2 polarization by multiple mechanisms.

The upregulation of certain genes including *Prl2c2*, *Prlc2c3*, *Snord32a*, *Snora74a*, *Oc-stamp* and *Car3* by *Notch1* haploinsufficiency in response to IL4/IL13 is particularly intriguing. Prl2c2 and Prl3c3 belong to the prolactin/somatotropin/placental lactogen family of peptide hormones. In a recent study, expression profiling of macrophages revealed upregulation of Prl2c2 and Prl3c3 and their association with M2-polariztion^56^. Recent studies have shown that small nucleolar RNAs (Snord and Snora) function as regulators of metabolic stress response pathways in mammalian cells and also have been implicated in cancer development and progression^57^. Oc-stamp is a RANKL-induced, multi-pass transmembrane protein that promotes the formation of multinucleated osteoclasts and is strongly upregulated in primary pre-osteoclasts^45^. Oc-stamp requires STAT6 and IL4 signaling for its activation^58^. Carbonic anhydrases are osteoclastogenic genes and have been associated with mannose receptor–positive M2 macrophages^59^. It will be interesting to determine if *Notch1* haploinsufficiency promotes M2 polarization of macrophages through these genes.

With *Notch1* haploinsufficiency, there was a downregulation of few genes to less than 50% of the WT BMDMs in the presence of IL4/IL13 including histidine ammonia lyase (*Hal*), *Sox18*, plexin A4 opposite strand 1 (*Plxna4os1*), *Oas2* and *Gpr31b*. Hal initiates the catabolism of anti-inflammatory histidine into histamine thus making the cells susceptible to pro-inflammatory factors and pro-oxidant^60^. Sox18 is a transcription factor critically required for tumor-induced lymphangiogenesis, and suppressing Sox18 function impedes tumor metastasis^61^. The instability of Notch1 and its susceptibility to degradation has been well reported in the literature^37,62–64^. In our RNA-Seq analysis, genes related to RNA degradation (Cnot1, Cnot6, Cnot6l, Lsm6, and Ttc37), RNA polymerase (Polr2a, Polr2f, Polr3b and Znrd1) and RNA transport (Cyfip2, Eif2b2, Kpnb1, Ndc1, Nxt1and Ranbp2) were dysregulated by *Notch1* haploinsufficiency suggesting its role in RNA degradation. Oas2 belong to members of RNA polymerase enzymes that initiate RNA destabilization through activation of RNase L^65,66^. It will be pertinent to examine the susceptibility of Notch1 mRNA transcript under various stress conditions and its impact on the downstream targets. It will be interesting to know if mutated Notch1 allele generate a truncated protein that is still active but has unexpected impact on transcription at the Notch 1 locus or stability of the Notch 1 mRNA transcript.

Using our novel algorithm approach along with existing knowledge from KEGG pathways and protein-protein interaction networks in mouse, our data provide *in silico* hypothesis to complement existing hypothesis as well as build new follow-up studies (Fig. 8). Differential effects of Notch deficiency (haploinsufficiency, Notch1-siRNA or DAPT) on its downstream effectors may be explained by partial deletion, off-target effects associated with siRNAs or lack of specificity of pharmacological inhibition. Moreover, the basal expression of these genes in the unstimulated and stimulated cells may also affect their differential response. The use of global *Notch1* haploinsufficient rather than myeloid-specific Notch1 knockout mice also reflect a limitation in our approach as other cells may influence the functionality and polarization of macrophages. Obviously, more studies will be required to explain these discrepancies. In conclusion, we provide evidence that Notch1 signaling plays a key role in M2-polarization of macrophages through Fmod- dependent regulation of Tgf-β2-signaling pathways. These traits of *Notch1* haploinsufficiency will open up new avenues to advance research in disease models involving these unique genes. Validation of these unique genes in a mouse model and human pathologies is warranted in the follow-up studies.

## Materials and Methods

### Macrophage isolation and treatments, RNA extraction and quality control

Six to eight week old *Notch1*^+/−^ male and *WT* female mice (C57BL/6 J background; Jackson Laboratory) were crossbred to generate *WT and Notch1*^+/−^ mice (n = 12 each). Generation of the C57BL/6 *Notch1*^+/−^ mice has been described previously^38^. Mice were kept on a 12 h/12 h light/dark cycle with standard chow. Genotyping was performed according to the protocol from Jackson Laboratories (Supplemental Fig. 1A). BMDMs were isolated from six to eight week old *WT* or *Notch1*^+/−^ littermates by previously established protocol^20,21^. Briefly, femur and tibia bones were flushed with culture media under aseptic conditions and cells were collected and treated with ACK lysis buffer (Gibco) to lyse RBCs. The remaining bone-marrow derived cells were cultured in ultra-low 6 well tissue culture plates. After 6 h, cells were treated with macrophage colony stimulating factor (M-CSF; 10 ng/ml, R&D systems) in complete medium (RPMI 1640 + 10% human serum + 1% penicillin streptomycin) with a media change every other day. At day 7, cells were serum starved in 1% FCS-RPMI for 2 h followed by stimulation with lipopolysaccharide (LPS; 100 ng/ml, Sigma Aldrich) and interferon-γ (IFN-γ; 20 ng/ml, Biolegend) or IL-4/IL-13 (10 ng/ml each, R&D systems) for 24 h to polarize bone-marrow derived cells into M1 or M2 states respectively^20,21,67^. For naïve macrophages, cells were treated with vehicle only. For gene expression analysis, media was removed and the cell pellets were mixed with RLT lysis buffer (Qiagen). Total RNA was extracted using the RNeasy kit (Qiagen) following the manufacturers’ instructions. Isolated RNA was further processed with DNase treatment and removal reagent (Ambion) to eliminate genomic DNA. The concentration of extracted RNA was measured using a Nanodrop ND1000 to verify that the 260/280 and 260/230 ratios were ∼2.

Samples were then stored at −80 °C until shipped to Ocean Ridge Biosciences LLC (Palm Beach Gardens, FL) for quality control (QC) analyses and RNA-Sequencing^68^. The RNA was quantified again by Ocean Ridge using O.D. measurement and assessed for quality on a 1% agarose – 2% formaldehyde RNA QC gel (Supplemental Fig. 1B)^68^. A portion of cDNA was used to confirm the downregulation of *Notch1* gene expression and its downstream targets in the *Notch1*^+/−^ BMDMs by qRT-PCR (Supplemental Fig. 1C). The samples were run in triplicate and the fold-change was determined by normalizing CT values against *Rpl13a*. The primers sequences used in our study are shown in Supplemental Table 3. The read counts in the region of the PCR primers aligning to Notch1 in the *WT* and *Notch1*^+/−^ BMDMs were analyzed for mapping quality and alignment and RNA degradation (Supplemental Fig. 1D–G).

### Library preparation, sequencing and data pre-processing

A total of 1000 ng of DNA-free total RNA was used as the input for a TruSeq Stranded mRNA Library Prep Kit (Illumina Inc., San Diego, CA) to prepare amplified cDNA libraries suitable for sequencing. The quality and size distribution of the amplified libraries were determined using an Agilent 2100 Bioanalyzer High Sensitivity DNA Chip. Libraries were quantified using the KAPA Library Quantification Kit (Kapa Biosystems, Boston, MA). The libraries were pooled at equimolar concentrations and diluted prior to loading onto the flow cell of the Illumina cBot cluster station. The libraries were extended and bridge amplified to create sequence clusters using the Illumina HiSeq PE Cluster Kit v4 and sequenced on an Illumina HiSeq Flow Cell v4 with 50-bp paired-end reads plus index read using the Illumina HiSeq SBS Kit v4. Real time image analysis and base calling were performed on the instrument using the HiSeq Sequencing Control Software version 2.2.58. All samples had a minimum of 51,304,689 passed-filter 50 nucleotide paired-end reads. The samples aligned at an average of 87.5% ± 0.5% (SD) efficiency to the reference genome. The raw fastq files were split into files containing 4,000,000 reads and checked for quality using fastx toolbox^69^. Based on the quality results, the reads were filtered (removing sequences that did not pass Illumina’s quality filter) and trimmed of 3 nucleotides at the left end of R1 and 1 nucleotide from the left end of R2.

### Sequence alignment, exon and gene level counting, annotation and filtering

Sequence alignment was performed using TopHat v2.1.0 using the Ensembl mouse reference genome release 83 and mm10 BOWTIE2 GENOME INDEX files from ftp://ftp.ccb.jhu.edu/pub/data/bowtie2_indexes/^70-72^. For exon and gene level counting Bioconductor easyRNASeq.^3^ v. 2.4.7 package running on R version 3.2.2 platform was used^73^. A binary annotation file (gAnnot_GRCm38.83.rda, see accompanying annotation folder) built using the annotation file generation function of easy RNASeq, was used for this analysis; the annotation version is Ensembl mouse release 83. The easyRNASeq software was downloaded from: http://www.bioconductor.org/packages/release/bioc/html/easyRNASeq.html.

A Gene Transfer Format (GTF) annotation file for Mus musculus was downloaded from Ensembl Biomart. This file, ensembl_Mus_musculus.GRCm38.83.chr.gtf, contained the current Ensembl Mouse Release 83. Descriptions for each gene in the Ensembl Mouse Release 83 GTF file were downloaded from Biomart and added to the annotation from the GTF file to generate the final file named mart83_export_mmu38-rev_052016.txt. This file contained 708,810 rows, which represent 448,141 unique exons and 46,983 unique genes. The RPKM values were filtered to retain a list of genes with a minimum of approximately 50 mapped reads in one or more samples. The threshold of 50 mapped reads is considered the Reliable Quantification Threshold, since the RPKM values for a gene represented by 50 reads should be reproducible in technical replicates. To avoid reporting large fold changes due to random variation of counts from low abundance mRNA, RPKM values equivalent to a count of < = 10 reads per gene were replaced with the average RPKM value equivalent to 10 reads/gene across all the samples in the experiment.

### WebGestalt analysis and validation by qRT-PCR and Immunostaining

WebGestalt software was utilized for a statistics-based pathway analysis in order to determine the distribution of differentially expressed genes (DEGs) among functional biological pathways^74^. The software compares the relative distribution of genes that met specific significance criteria to the distribution of all detectable genes. Statistical significance in specific pathways is based on an adjusted *p* < *0*.05 for enrichment of genes meeting the selection criteria, relative to the reference genes. The WebGestalt software was used to query three pathway databases including KEGG, Wiki Pathways, and GO Pathways^34,75^.

For the validation of DEGs, naïve macrophages were collected from the BMDMs of *WT* or *Notch1*^+/−^ mice (triplicate; pooled from 9 mice each) as detailed earlier^20,21^. Naïve macrophages at day 7 or RAW cells (264.7, a murine monocyte/macrophage cell line, ATCC) were pre-treated with DAPT (10 μM) or DMSO for 72 h prior to incubation with LPS/IFN-γ or IL4/IL13 as described^76^. Following treatment, RAW cells and macrophages were washed with cold PBS and RNA extracted using RNAeasy kit (Qiagen). cDNA was synthesized using SuperScript VILO™ cDNA Synthesis Kit (Invitrogen) and subjected to qRT-PCR by SYBR Green RT-PCR kit (BioRad) using Applied Biosystems 7500 Fast Real-Time PCR or Biorad CFX Connect™. The samples were run in triplicate and the fold-change was determined by normalizing CT values against *Rpl13a*. DIF staining of BMDMs was performed using Fmod and Tgf-β2 antibodies, nuclei were stained with DAPI. Quantification of DIF was performed using Lionheart FX gen5 software^77^. To determine the stability of Notch1 mRNA, macrophages were treated with Actinomycin D (20 µg/ml, A1410; Sigma) or human recombinant HuR (10 ng/ml, Novus Biologicals) for 24 h and RNA was extracted using RNAeasy kit (Qiagen).

### Transfection of BMDMs and FMOD treatments

Plasmids and siRNA sequences were used for transfecting macrophages. NICD plasmids were obtained from Dr. Lilly’s laboratory^19,78^. Predesigned Notch1 (s70698, Invitrogen) siRNA were used with Lipofectamine 3000 (L3000, Invitrogen) and Lipofectamine RNA iMAX (13778, Invitrogen) as explained^19,78^. The cells were plated into 6-well plates with normal growth serum. Two hours prior to transfection, DMEM media without serum and antibiotics were added to the cells. The reagents for transfection were prepared using OptiMEM media (31985-070, Invitrogen). Two different tubes were prepared for each well; one containing the OptiMEM and Lipofectamine P3000 and the other containing OptiMEM, Lipofectamine P3000/RNA iMAX reagent, and either the plasmid (2 µl/µg DNA) or the siRNA for Notch1. These two tubes were then combined and the mixture was incubated for 5 minutes at room temperature. This mixture was then added directly onto the cells containing the DMEM serum-free and antibiotic-free media. The media was changed four to six hours after transfection with normal growth medium. RNA was extracted 48 h post transfection and subjected to qRT-PCR. To determine the effect of FMOD on M1 and M2 genes, macrophages were pre-treated in serum-free RPMI culture medium followed by treatment with recombinant human FMOD Protein (400 ng/ml; Ab152392, Abcam) for 24 h.

### FMOD and MMP8 treatments, protein isolation, Western blot and Quantification

In a separate experiment, macrophages were pre-treated overnight with DMSO or DAPT (10 μM) in serum-free RPMI culture medium followed by treatment with either Recombinant Human FMOD Protein (400 ng/ml; Ab152392, Abcam) or activated-MMP8 (400 ng/ml, Fisher) for 24 h. MMP8 was activated using p-aminophenylmercuric acetate (APMA) following a standard protocol. RNA was extracted from these cells for gene-expression study. In some wells, BMDMs from WT or *Notch1*^+/−^ were treated with either TGF-β2 (human recombinant protein; 5 ng/ml, Fisher), TGFβ receptor kinase inhibitor (SB‐431542; 15 nM, Tocris Biosciences), FMOD (100 ng/ml) or MMP8 inhibitor (400 ng/ml, Fisher) for 24 h. The media was concentrated using Amicon® Ultra-4 Centrifugal Filter Units (3 KDa, Millipore) to 100 μl (1:50) and Western blot was performed to detect secreted Tgf-β2 (as a measure of active-Tgf-β signaling) using a standard protocol^76,79–81^.

Total proteins were extracted from the cells with RIPA buffer supplemented with protease and phosphatase inhibitor cocktails (Roche). After homogenization, proteins concentrations were measured using a Pierce^tm^ BCA Protein Assay Kit (Thremo-Fisher) and 10 µg of protein was subsequently loaded onto a 10% SDS-polyacrylamide gel and transferred to PVDF membrane using standard protocols^19,80,82^. The membranes were probed for Fmod (Ab81443, Abcam), TGF-β2 (Ab36495, Abcam), MMP8 (Ab81286, Abcam) and NICD (Ab8925, Abcam) following standard protocols^76,79–81^. GAPDH (NB300-221, Novus Biologicals) and Coomassie Blue staining (Bio-Rad) were used as the internal loading controls for the cells and media respectively. Western blots were quantified using Image J software in three replicates and representative blot is shown^83^.

### Isolation of peritoneal macrophages and Co-Immunoprecipitation

Peritoneal macrophages from WT and *Notch1*^+/−^ mice were isolated three days after thioglycollate stimulation as described previously^80^. The anti-Fmod antibody (100 µg) were coupled with dynabeads (5 mg) overnight following the recommended protocol (14321D, life technologies). The cell lysates derived from WT and *Notch1*^+/−^ peritoneal macrophages were pulled down with FMOD antibody. The Co-IP products were washed three times with PBS. After the final wash, the dynabeads were re-suspended in 40 μL of sample buffer were probed for TGF-β2, Fmod and NICD by Western blot.

### Study Approval

All animal-related experiments were approved by the Institutional Animal Care and Use Committee at the Research Institute at Nationwide Children’s Hospital (Columbus, OH) and at the University of Missouri (Columbia, MO). All the methods were performed in accordance with the relevant guidelines and regulations. The IF and Western blot images represent the original data and conform to community standards.

### KBCommons database for mouse

The analyzed gene expression datasets and gene modules can be accessed interactively via mouseKB in KBCommons (http://kbcommons.org/system/browse/diff_exp/MusMusculus), a comprehensive framework fully equipped with database and informatics tools for multi-omics data analysis. It provides a set of visualization and analytical tools such as differential expression analysis and gene card pages and provides data in the form of tabs for Gene lists, Venn diagram, Volcano plot, Function Analysis, Pathway Analysis and Gene modules. The Function Analysis tab provides a functional annotation pie chart and particular annotation lists for gene families and PFAM domains^84^. The Pathway Analysis tab retrieves the KEGG^8^ pathway mapping for the genes^34^. The Gene module tab provides weighted correlation network analysis (WGCNA) which identifies the correlation patterns among genes and forms modules based on that analysis^31^.

### Statistical analysis

Pearson’s correlation coefficient using log_2_ and 2-way ANOVA using replicate pairs was performed on the RPKM data for the 20,375 detectable mouse genes to examine the effect of cell type (*WT* and *Notch1*^+/−^) or treatments (Control, LPS/IFN-γ, and IL4/IL13) as well as their interactions on gene expression. Tukey’s tests were performed to determine the effects of treatment against control within each cell type, and the effect of cell type at each treatment. Fold changes were calculated for the same comparisons by comparing the mean values from both groups. If the mean of both groups under comparison were below the Detection Threshold (10 reads/ gene), “NA” was reported. All statistical analyses were performed using R version 3.2.2 statistical computing software. For the real-time qRT-PCR quantification, we performed the Kruskal–Wallis test using a non-parametric method for the overall significance of the data and ordinary ANOVA followed by a Turkey’s multiple comparisons test using GraphPad Prism version 5.0. The additional datasets generated during in the current study are available from the corresponding author on reasonable request.

## Supplementary information

Dataset 1

## Data Availability

For the original RNASeq data files, contact Dr. Chetan Hans. For further information about bioinformatics, contact Trupti Joshi. The analyzed gene expression datasets and gene modules can be accessed interactively via mouseKB in KBCommons (http://kbcommons.org/system/browse/diff_exp/MusMusculus), a comprehensive framework fully equipped with database and informatics tools for multi-omics data analysis.

## References

[1] El-Gabalawy H, Guenther LC, Bernstein CN (2010). Epidemiology of immune-mediated inflammatory diseases: incidence, prevalence, natural history, and comorbidities. J Rheumatol Suppl.

[2] Motwani MP, Gilroy DW (2015). Macrophage development and polarization in chronic inflammation. Semin Immunol.

[3] Prud’homme GJ (2007). Pathobiology of transforming growth factor beta in cancer, fibrosis and immunologic disease, and therapeutic considerations. Lab Invest.

[4] Vujosevic S, Simo R (2017). Local and Systemic Inflammatory Biomarkers of Diabetic Retinopathy: An Integrative Approach. Invest Ophthalmol Vis Sci.

[5] Sica A, Erreni M, Allavena P, Porta C (2015). Macrophage polarization in pathology. Cell Mol Life Sci.

[6] Gaffney, L., Warren, P., Wrona, E. A., Fisher, M. B. & Freytes, D. O. In *Macrophages: Origin*, *Functions and* Biointervention (ed Malgorzata Kloc) 245–271 (Springer International Publishing, 2017).

[7] Valledor, A. F., Comalada, M., Santamaría-Babi, L. F., Lloberas, J. & Celada, A. In *Advances in Immunology* Vol. 108 (eds Frederick W. Alt *et al*.) 1–20 (Academic Press, 2010). 10.1016/B978-0-12-380995-7.00001-X 21056727

[8] Chinetti-Gbaguidi G, Staels B (2011). Macrophage polarization in metabolic disorders: functions and regulation. Curr Opin Lipidol.

[9] Xu L, Kitade H, Ni Y, Ota T (2015). Roles of Chemokines and Chemokine Receptors in Obesity-Associated Insulin Resistance and Nonalcoholic Fatty Liver Disease. Biomolecules.

[10] Novak ML, Koh TJ (2013). Phenotypic transitions of macrophages orchestrate tissue repair. Am J Pathol.

[11] Zhou D (2017). Macrophage polarization and function: new prospects for fibrotic disease. Immunol Cell Biol.

[12] Vergadi E, Ieronymaki E, Lyroni K, Vaporidi K, Tsatsanis C (2017). Akt Signaling Pathway in Macrophage Activation and M1/M2 Polarization. J Immunol.

[13] Murray PJ (2017). Macrophage Polarization. Annual Review of Physiology.

[14] Jablonski KA (2015). Novel Markers to Delineate Murine M1 and M2 Macrophages. PloS one.

[15] Martinez FO, Gordon S (2014). The M1 and M2 paradigm of macrophage activation: time for reassessment. F1000Prime Reports.

[16] Murray PJ (2014). Macrophage activation and polarization: nomenclature and experimental guidelines. Immunity.

[17] Nakano T, Fukuda D, Koga J, Aikawa M (2016). Delta-Like Ligand 4-Notch Signaling in Macrophage Activation. Arterioscler Thromb Vasc Biol.

[18] Xu J, Chi F, Tsukamoto H (2015). Notch signaling and M1 macrophage activation in obesity-alcohol synergism. Clin Res Hepatol Gastroenterol.

[19] Sachdeva J (2017). Smooth muscle cell-specific Notch1 haploinsufficiency restricts the progression of abdominal aortic aneurysm by modulating CTGF expression. PloS one.

[20] Cheng J, Koenig SN, Kuivaniemi HS, Garg V, Hans CP (2014). Pharmacological inhibitor of notch signaling stabilizes the progression of small abdominal aortic aneurysm in a mouse model. J Am Heart Assoc.

[21] Hans CP (2012). Inhibition of Notch1 signaling reduces abdominal aortic aneurysm in mice by attenuating macrophage-mediated inflammation. Arterioscler Thromb Vasc Biol.

[22] Singla DK, Wang J, Singla R (2017). Primary human monocytes differentiate into M2 macrophages and involve Notch-1 pathway. Can J Physiol Pharmacol.

[23] Xu H (2012). Notch-RBP-J signaling regulates the transcription factor IRF8 to promote inflammatory macrophage polarization. Nat Immunol.

[24] Zhou D (2014). Macrophage polarization and function with emphasis on the evolving roles of coordinated regulation of cellular signaling pathways. Cell Signal.

[25] Hu, Y.-Y., Zheng, M.-h., Zhang, R., Liang, Y.-M. & Han, H. In *Notch Signaling in Embryology and Cancer* Vol. 727 *Advances in Experimental Medicine and Biology* (eds Jörg Reichrath & Sandra Reichrath) Ch. 14, 186–198 (Springer US, 2012).

[26] Gridley T (2010). Notch signaling in the vasculature. Curr Top Dev Biol.

[27] Bray SJ (2006). Notch signalling: a simple pathway becomes complex. Nat Rev Mol Cell Biol.

[28] Quillard T, Charreau B (2013). Impact of notch signaling on inflammatory responses in cardiovascular disorders. Int J Mol Sci.

[29] Zou S (2012). Notch signaling in descending thoracic aortic aneurysm and dissection. PloS one.

[30] de Hoon MJ, Imoto S, Nolan J, Miyano S (2004). Open source clustering software. Bioinformatics.

[31] Langfelder P, Horvath S (2008). WGCNA: an R package for weighted correlation network analysis. BMC Bioinformatics.

[32] Sharov AA, Dudekula DB, Ko MS (2005). A web-based tool for principal component and significance analysis of microarray data. Bioinformatics.

[33] Ashburner M (2000). Gene ontology: tool for the unification of biology. The Gene Ontology Consortium. Nat Genet.

[34] Kanehisa M, Sato Y, Kawashima M, Furumichi M, Tanabe M (2016). KEGG as a reference resource for gene and protein annotation. Nucleic Acids Res.

[35] Cisneros E, Latasa MJ, García-Flores M, Frade JM (2008). Instability of Notch1 and Delta1 mRNAs and reduced Notch activity in vertebrate neuroepithelial cells undergoing S-phase. Molecular and Cellular Neuroscience.

[36] Houseley J, Tollervey D (2009). The Many Pathways of RNA Degradation. Cell.

[37] Wang Z (2017). Stabilization of Notch1 by the Hsp90 Chaperone is Crucial for T-Cell Leukemogenesis. Clinical Cancer Research.

[38] Conlon RA, Reaume AG, Rossant J (1995). Notch1 Is Required for the Coordinate Segmentation of Somites. Development.

[39] Wen G (2015). A Novel Role of Matrix Metalloproteinase-8 in Macrophage Differentiation and Polarization. J Biol Chem.

[40] Shami A (2013). Fibromodulin deficiency reduces low-density lipoprotein accumulation in atherosclerotic plaques in apolipoprotein E-null mice. Arterioscler Thromb Vasc Biol.

[41] Awad F (2017). Impact of human monocyte and macrophage polarization on NLR expression and NLRP3 inflammasome activation. PloS one.

[42] Kim EY, Kim BC (2011). Lipopolysaccharide inhibits transforming growth factor-beta1-stimulated Smad6 expression by inducing phosphorylation of the linker region of Smad3 through a TLR4-IRAK1-ERK1/2 pathway. FEBS Lett.

[43] Mitchell K (2014). LPS antagonism of TGF-beta signaling results in prolonged survival and activation of rat primary microglia. J Neurochem.

[44] Jan AT, Lee EJ, Choi I (2016). Fibromodulin: A regulatory molecule maintaining cellular architecture for normal cellular function. Int J Biochem Cell Biol.

[45] Yang M (2008). Osteoclast stimulatory transmembrane protein (OC-STAMP), a novel protein induced by RANKL that promotes osteoclast differentiation. J Cell Physiol.

[46] Ito R (2012). Osteosclerosis and inhibition of human hematopoiesis in NOG mice expressing human Delta-like 1 in osteoblasts. Exp Hematol.

[47] Nakajima K (2014). Galectin-3 inhibits osteoblast differentiation through notch signaling. Neoplasia.

[48] Hu M (2014). Notch signaling regulates col1alpha1 and col1alpha2 expression in airway fibroblasts. Exp Biol Med (Maywood).

[49] Wilkinson TS, Bressler SL, Evanko SP, Braun KR, Wight TN (2006). Overexpression of hyaluronan synthases alters vascular smooth muscle cell phenotype and promotes monocyte adhesion. J Cell Physiol.

[50] Woodruff PG (2005). A distinctive alveolar macrophage activation state induced by cigarette smoking. Am J Respir Crit Care Med.

[51] John AE (2016). Loss of epithelial Gq and G11 signaling inhibits TGFbeta production but promotes IL-33-mediated macrophage polarization and emphysema. Sci Signal.

[52] Abe Y (2013). TIM-4 has dual function in the induction and effector phases of murine arthritis. J Immunol.

[53] Lakshmanan U, Porter AG (2007). Caspase-4 Interacts with TNF Receptor-Associated Factor 6 and Mediates Lipopolysaccharide-Induced NF- B-Dependent Production of IL-8 and CC Chemokine Ligand 4 (Macrophage-Inflammatory Protein-1). The Journal of Immunology.

[54] Kushiyama T (2011). Alteration in the phenotype of macrophages in the repair of renal interstitial fibrosis in mice. Nephrology (Carlton).

[55] Riquelme P (2017). Dhrs9 Is a Specific and Stable Marker of Human Regulatory Macrophages. Transpl Int.

[56] Poczobutt JM (2016). Expression Profiling of Macrophages Reveals Multiple Populations with Distinct Biological Roles in an Immunocompetent Orthotopic Model of Lung Cancer. J Immunol.

[57] Romano G, Veneziano D, Acunzo M, Croce CM (2017). Small non-coding RNA and cancer. Carcinogenesis.

[58] Miyamoto H (2012). An essential role for STAT6-STAT1 protein signaling in promoting macrophage cell-cell fusion. J Biol Chem.

[59] Chinetti-Gbaguidi G (2017). Human Alternative Macrophages Populate Calcified Areas of Atherosclerotic Lesions and Display Impaired RANKL-Induced Osteoclastic Bone Resorption Activity. Circulation research.

[60] Calve S, Isaac J, Gumucio JP, Mendias CL (2012). Hyaluronic acid, HAS1, and HAS2 are significantly upregulated during muscle hypertrophy. American journal of physiology. Cell physiology.

[61] Dabritz J (2015). Reprogramming of monocytes by GM-CSF contributes to regulatory immune functions during intestinal inflammation. J Immunol.

[62] Kim M-Y (2011). Regulation of Notch1 signaling by the APP intracellular domain facilitates degradation of the Notch1 intracellular domain and RBP-Jk. Journal of cell science.

[63] Lim J, Lee K-m, Shim J, Shin I (2014). CD24 regulates stemness and the epithelial to mesenchymal transition through modulation of Notch1 mRNA stability by p38MAPK. Archives of Biochemistry and Biophysics.

[64] McGill MA, Dho SE, Weinmaster G, McGlade CJ (2009). Numb Regulates Post-endocytic Trafficking and Degradation of Notch1. The Journal of Biological Chemistry.

[65] Kristiansen H, Gad HH, Eskildsen-Larsen S, Despres P, Hartmann R (2011). The oligoadenylate synthetase family: an ancient protein family with multiple antiviral activities. J Interferon Cytokine Res.

[66] Choi UY, Kang JS, Hwang YS, Kim YJ (2015). Oligoadenylate synthase-like (OASL) proteins: dual functions and associations with diseases. Exp Mol Med.

[67] Weischenfeldt J, Porse B (2008). Bone Marrow-Derived Macrophages (BMM): Isolation and Applications. CSH Protoc.

[68] Barnette DN, VandeKopple M, Wu Y, Willoughby DA, Lincoln J (2014). RNA-seq analysis to identify novel roles of scleraxis during embryonic mouse heart valve remodeling. PloS one.

[69] Lab, H.

[70] Trapnell C, Pachter L, Salzberg SL (2009). TopHat: discovering splice junctions with RNA-Seq. Bioinformatics.

[71] Langmead B, Trapnell C, Pop M, Salzberg SL (2009). Ultrafast and memory-efficient alignment of short DNA sequences to the human genome. Genome Biol.

[72] Kim D, Salzberg SL (2011). TopHat-Fusion: an algorithm for discovery of novel fusion transcripts. Genome Biol.

[73] Delhomme N, Padioleau I, Furlong EE, Steinmetz L (2012). M. easyRNASeq: a bioconductor package for processing RNA-Seq data. Bioinformatics.

[74] Wang J, Duncan D, Shi Z, Zhang B (2013). WEB-based GEne SeT AnaLysis Toolkit (WebGestalt): update 2013. Nucleic Acids Res.

[75] Gene Ontology C (2015). Gene Ontology Consortium: going forward. Nucleic Acids Res.

[76] Acharya A (2011). Inhibitory role of Notch1 in calcific aortic valve disease. PloS one.

[77] Kalidhindi, R. S. R. *et al*. In *D58*. *Airways Hyperresponsiveness*: *Novel Mechanisms and Targets American Thoracic Society International Conference Abstracts* A7266–A7266 (American Thoracic Society, 2018).

[78] Lin CH, Lilly B (2014). Notch signaling governs phenotypic modulation of smooth muscle cells. Vascul Pharmacol.

[79] Bosse K (2013). Endothelial nitric oxide signaling regulates Notch1 in aortic valve disease. J Mol Cell Cardiol.

[80] Oumouna-Benachour K (2007). Poly(ADP-ribose) polymerase inhibition reduces atherosclerotic plaque size and promotes factors of plaque stability in apolipoprotein E-deficient mice: effects on macrophage recruitment, nuclear factor-kappaB nuclear translocation, and foam cell death. Circulation.

[81] Zerfaoui M (2008). Nuclear translocation of p65 NF-kappaB is sufficient for VCAM-1, but not ICAM-1, expression in TNF-stimulated smooth muscle cells: Differential requirement for PARP-1 expression and interaction. Cell Signal.

[82] Hans CP (2009). Thieno[2,3-c]isoquinolin-5-one, a potent poly(ADP-ribose) polymerase inhibitor, promotes atherosclerotic plaque regression in high-fat diet-fed apolipoprotein E-deficient mice: effects on inflammatory markers and lipid content. J Pharmacol Exp Ther.

[83] Schneider CA, Rasband WS, Eliceiri KW (2012). NIH Image to ImageJ: 25 years of image analysis. Nat Methods.

[84] Punta M (2012). The Pfam protein families database. Nucleic Acids Res.

