# Supplementary material for: Transcriptomics Analysis Reveals New Insights into the Roles of Notch1 Signaling on Macrophage Polarization
Source: Sci Rep. 2019 May 29;9:7999. doi: 10.1038/s41598-019-44266-4 (PMC6541629; doi:10.1038/s41598-019-44266-4)
Supplement: Supplementary file 1 — Dataset 1 [file 41598_2019_44266_MOESM1_ESM.pdf]

Supplementary Material

Transcriptomics Analysis Reveals New Insights into the Roles of Notch1 Signaling on Macrophage Polarization

Chetan P. Hans[1,2,3*], Neekun Sharma[1,3], Sidharth Sen[4], Shuai Zeng[5], Rishabh Dev[1,3], Yuexu Jiang[5,6], Advitiya Mahajan[1], Trupti Joshi[4,5,7,8]

[1]Department of Cardiovascular Medicine & [2]Medical Pharmacology and Physiology; [3]Dalton Cardiovascular Research Center; [4]MU Informatics Institute; [5]Department of Computer Science; [6]Department of Electrical Engineering and Computer Science; [7]Department of Health Management and Informatics, School of Medicine; [8]Christopher S. Bond Life Science Center; University of Missouri, Columbia.

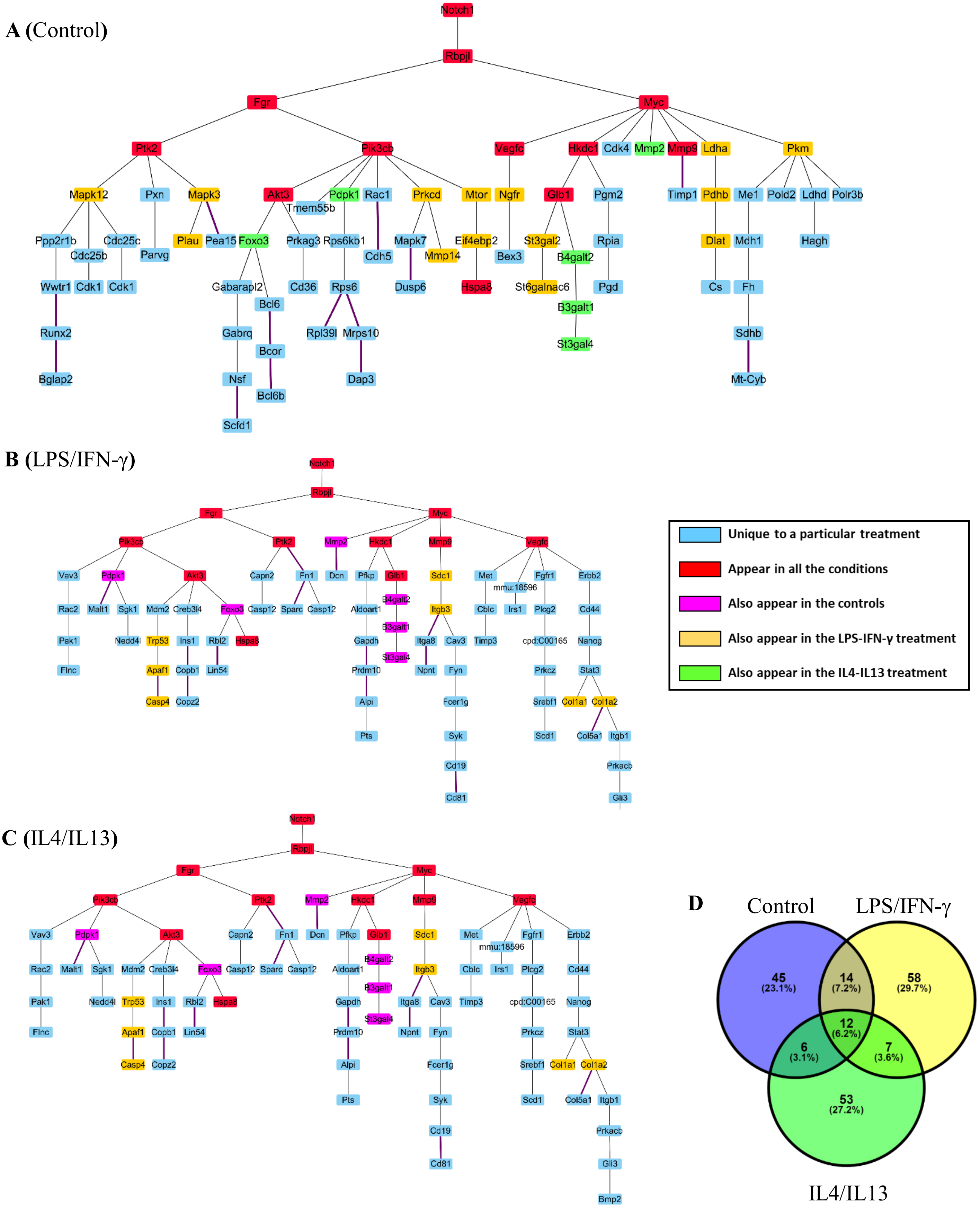

Supplemental Figure 1: Novel algorithm utilizing a existing knowledge from KEGG pathways and protein-protein interaction networks showing tree structures with Notch as the root of the tree for the novels pathways by which these genes may be regulated.

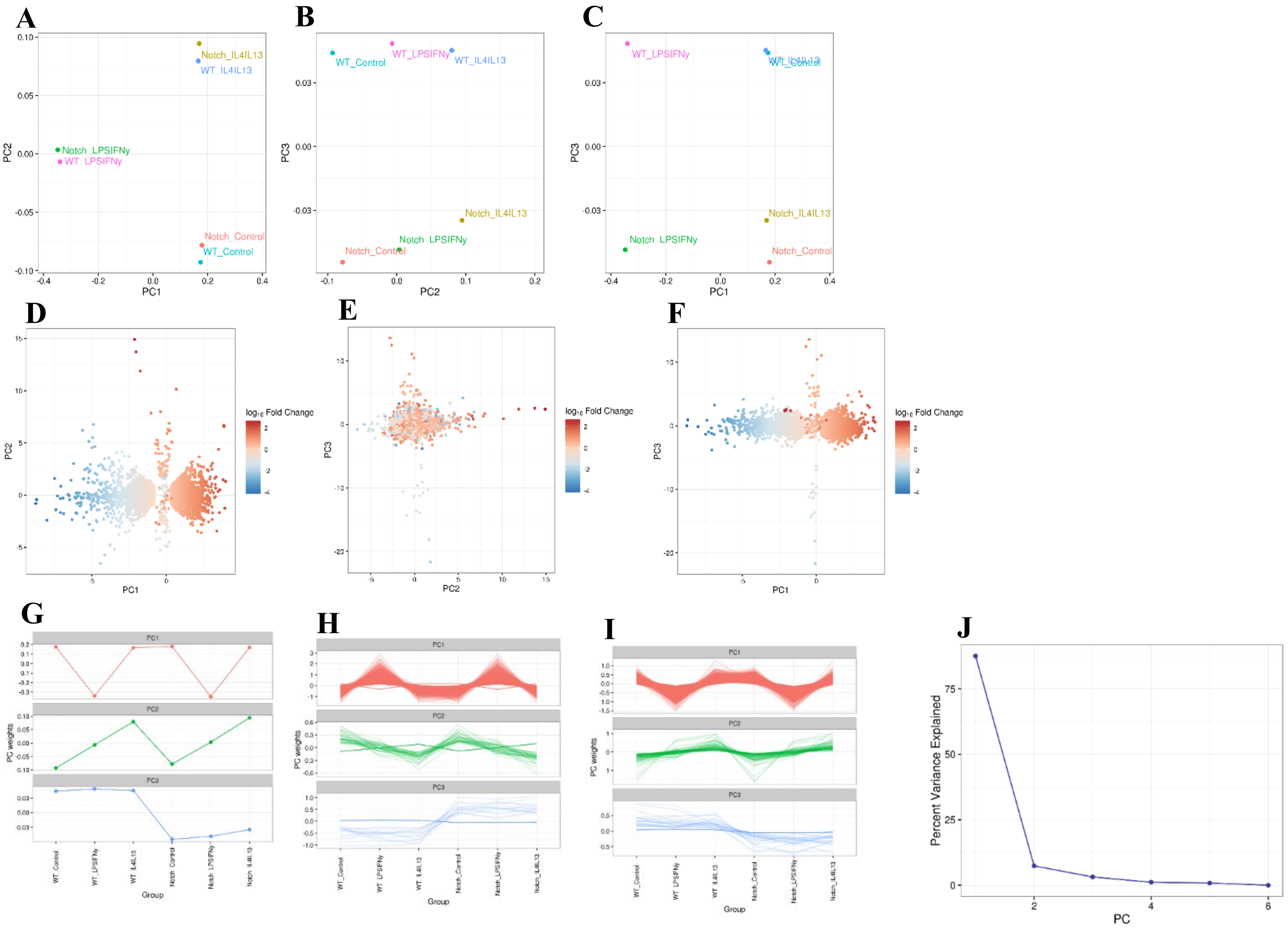

<u>**Supplemental Figure 2**</u>**:** Principle component analysis of gene expression displaying the expression trends within the data set. **(A-C)** Separation of samples based on their PC composite values. Points are colored according to the group name, and the color scale is arbitrary. **(D-F)** Separation of genes based on their PC composite values. Only genes meeting the cluster criteria are shown. **(G)** Sample-specific PC Plots. RPKM for individual genes making up their respective principal components are shown in light color. Overall value for PC composite values are shown in bold color. **(H-I)** Genes correlated with positive or negative principal component. **(J)** Screeplot of the percent variance explained versus principal component number. To find genes that substantially contribute to each PC value, the correlation and fold-change in expression of each gene with the first three principal components was calculated. Genes with a positive or negative correlation of at least 0.9 and a fold change of at least 2.0 compared with the principal components are reported.

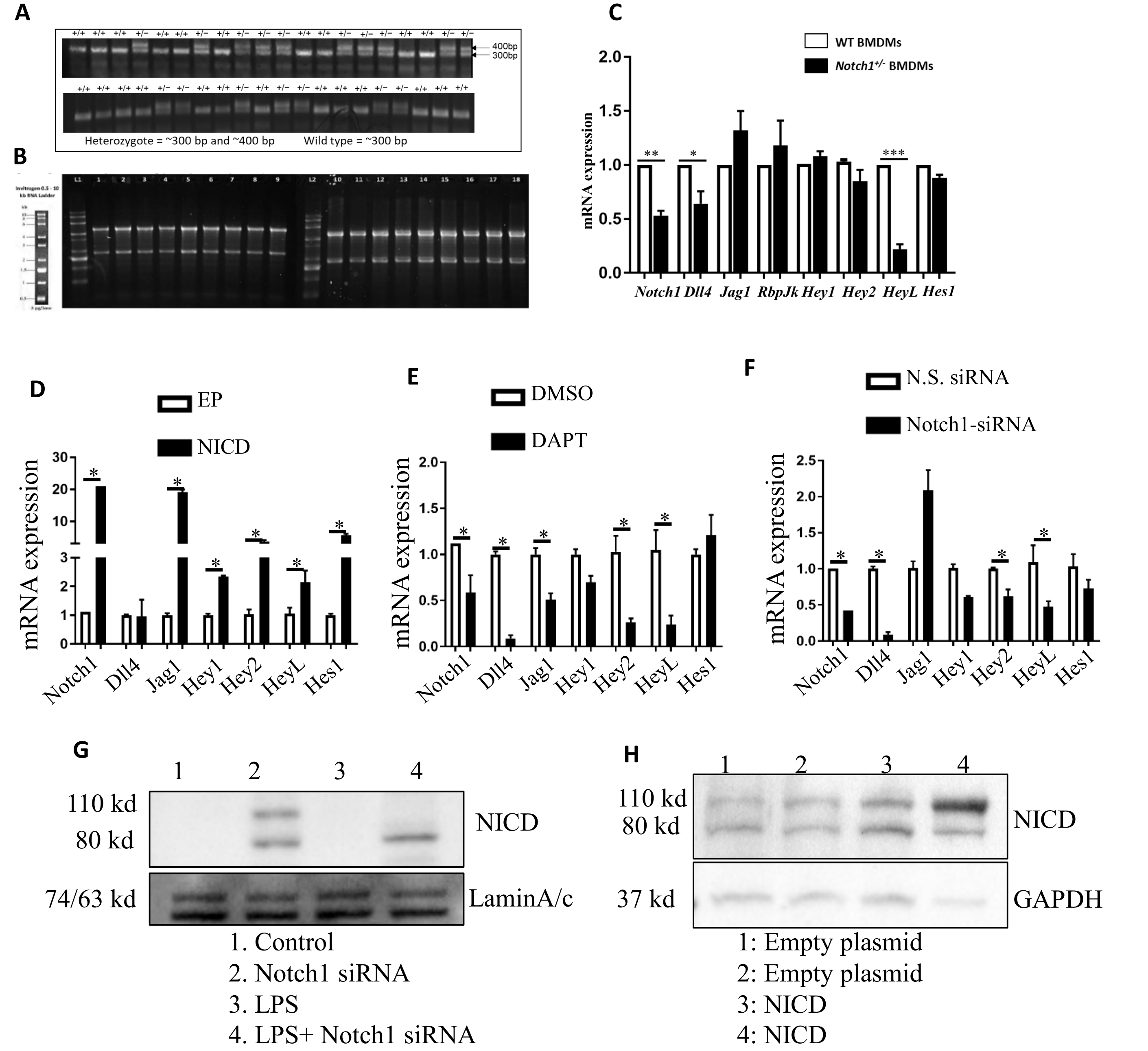

Supplemental Figure 3: Generation of *Notch1* haploinsufficient mice **(A)** Representative of genotype results presenting *Notch1*$^{+/-}$ haploinsufficient mice (double band with 400 and 300 bp) or WT mice (single band with 300 bp). **(B)** Quality assessment on a 1% agarose – 2% formaldehyde gel. **(C)** Bar graphs represent fold change in gene expression of *Notch1, HeyL* and *Dll4* in *Notch1*$^{+/-}$ BMDMs than *WT* BMDMs at basal levels. (**D**-**F**) mRNA expression of various Notch signaling pathway genes in RAW macrophages transfected with empty or NICD plasmid (D), treated with DMSO or Notch inhibitor, DAPT (E), and transfected with non-specific or Notch1 siRNA (F). **(G)** Western blot showing the protein content of NICD in the RAW cells treated with either control or Notch 1 specific siRNA in the absence and presence of LPS. (**H**) Western blot showing the protein content of NICD in the RAW cells transfected with either empty or NICD plasmids. Data are shown in duplicates. *** $p < 0.001$, ** $p < 0.01$, * $p < 0.05$.

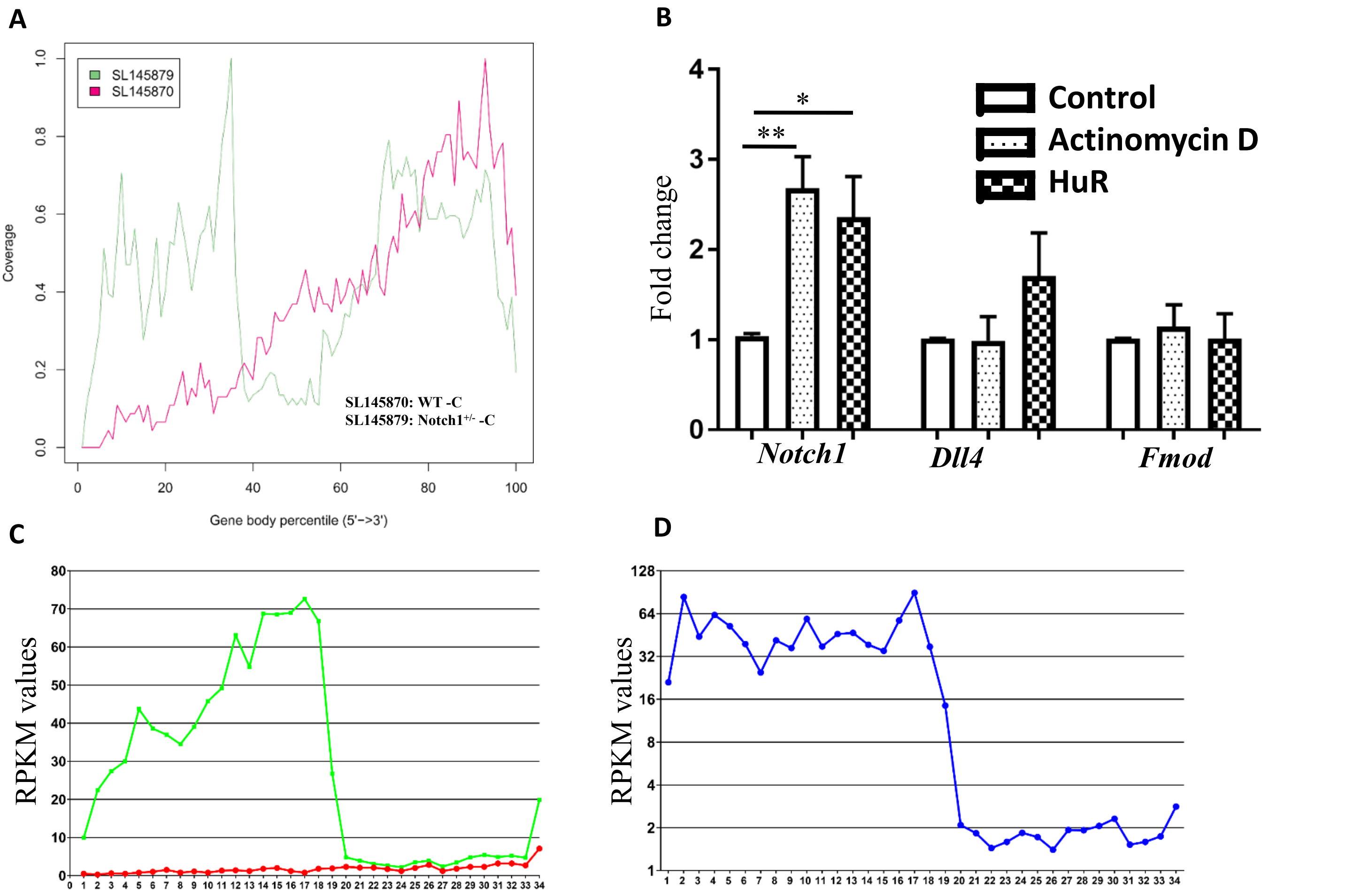

Supplemental Figure 4: (A) Read counts for *Notch1*$^{+/-}$ and *WT* samples. **(B)** RNA Stabilizers (Actinomycin D (10 µg/ml, or human recombinant HuR (100 ng/ml) increased the expression of Notch1 in BMDMs without affecting the expression of *Dll4* or *Fmod*. **(C)** shows the mean Notch1 RPKM values for the *WT* and *Notch1*$^{+/-}$ BMDMs for each exon in the full-length transcript. **(D)** shows the ratio of the mean RPKM values for *Notch1*$^{+/-}$ vs. WT. Note that the expression of Notch 1 mRNA is 30-60-fold higher in the *Notch1*$^{+/-}$ vs. WT only for the undeleted first 19 exons, consistent with dramatic up-regulation of the mutated Notch1 allele. **** $p < 0.001$, ** $p < 0.01$, * $p < 0.05$.*

A

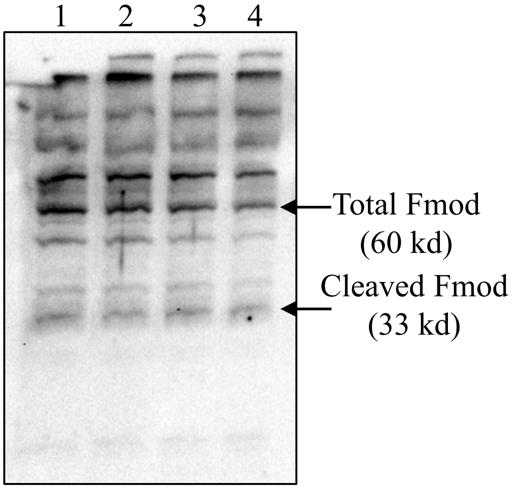

1. Empty plasmid
2. Empty plasmid
3. NICD
4. NICD

B

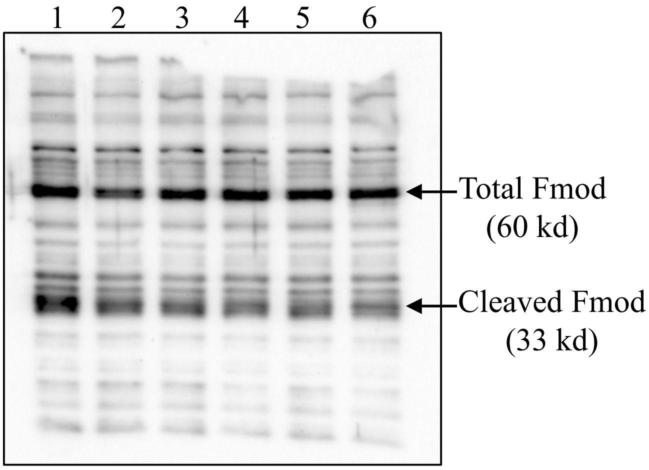

1. Control
2. LPS/IFN-γ -5 ng/ml
3. LPS/IFN-γ -10 ng/ml
4. LPS/IFN-γ -25 ng/ml
5. LPS/IFN-γ -50 ng/ml
6. LPS/IFN-γ -100 ng/ml

Supplemental Figure 5: (A) Full WB showing the expression of total and cleaved Fmod in *WT* BMDMs 48h post transfection with empty or NICD plasmid. (**B**) Full WB showing the contents of total and cleaved Fmod in macrophages in response to increasing dose of LPS (0, 5, 10, 25, 50 or 100 ng/ml) for 24h.

Supplemental Table 1: *List of top genes dysregulated by LPS/IFN-γ*

Fold Change: up > 2 fold
Fold Change: down < 0.5 fold

S. No.	Gene ID	Gene Name	WT LPS/IFN-γ vs WT-Mφ	Notch1+/- LPS/IFN-γ vs Notch1+/- Mφ
1	17329	Cxcl9	26243.32	15172.68
2	18126	Nos2	14898.17	35964.89
3	16365	Irg1	8960.42	6616.27
4	19225	Ptgs2	7256.59	2268.96
5	15945	Cxcl10	7213.04	6245.32
6	16193	Il6	4387.86	4768.75
7	20304	Ccl5	3391.98	4754.51
8	13421	Dnase1l3	3288.47	3622.68
9	20210	Saa3	2817.02	2268.62
10	17472	Gbp4	2506.48	1659.34
11	20299	Ccl22	2199.95	2017.01
12	238393	Serpina3f	2021.20	2498.68
13	16160	Il12b	1959.08	4016.97
14	229898	Gbp5	1741.78	2242.01
15	12515	Cd69	1595.76	2054.14
16	60440	Iigp1	1494.22	1697.15
17	231655	Oasl1	1106.89	3758.70
18	14289	Fpr2	1026.45	898.70
19	109979	Art3	1023.14	1101.72
20	326623	Tnfsf15	990.52	642.62
21	24108	Ubd	906.18	717.80
22	21822	Tgtp1	904.47	1242.62
23	20293	Ccl12	875.29	673.83
24	16176	Il1b	873.11	1199.94
25	13011	Cst7	869.13	1111.09
26	14825	Cxcl1	845.78	284.09
27	56066	Cxcl11	779.22	550.88
28	626578	Gbp10	772.37	823.51
29	20310	Cxcl2	750.98	680.95
30	16161	Il12rb1	716.27	463.93
31	14962	Cfb	667.71	516.41
32	634650	Gbp11	667.59	513.69
33	100702	Gbp6	661.31	886.68
34	80885	Hcar2	657.11	711.28
35	64380	Ms4a4c	566.50	1319.13
36	17167	Marco	518.59	246.42
37	20555	Slfn1	482.35	953.07
38	12810	Coch	474.72	618.18
39	623121	Pydc4	469.02	867.32
40	12775	Ccr7	460.98	369.44
41	224796	Clic5	451.55	746.93
42	14990	H2-M2	431.79	264.50
43	20287	Sct	403.29	164.53
44	14468	Gbp2b	400.89	644.90
45	12349	Car2	384.47	816.98
46	99899	Ifi44	366.79	1092.65
47	12266	C3	337.74	260.37
48	20714	Serpina3k	335.78	221.05
49	22271	Upp1	334.34	411.95
50	17857	Mx1	328.68	1035.83

S. No.	Gene ID	Gene Name	WT LPS/IFN-γ vs WT-Mφ	Notch1+/- LPS/IFN-γ vs Notch1+/- Mφ
1	64214	Rgs18	0.01	0.01
2	71738	Mamdc2	0.01	0.01
3	17179	Matk	0.01	0.01
4	76408	Abcc3	0.01	0.02
5	71085	Arhgap19	0.02	0.01
6	22403	Wisp2	0.02	0.01
7	140483	Hnmt	0.02	0.03
8	107585	Dio3	0.02	0.02
9	18054	Ngp	0.02	0.05
10	381810	Lpar5	0.02	0.01
11	72324	Plxdc1	0.02	0.01
12	11565	Adssl1	0.02	0.02
13	19716	Bex1	0.02	0.04
14	19400	Rapsn	0.02	0.03
15	406217	Bex4	0.02	0.05
16	12097	Bglap2	0.02	0.04
17	12487	Cd28	0.02	0.01
18	140742	Sesn1	0.02	0.02
19	12096	Bglap	0.02	0.04
20	71706	Slc46a3	0.02	0.02
21	19016	Pparg	0.02	0.04
22	75744	Svip	0.02	0.04
23	194590	Reps2	0.02	0.03
24	21953	Tnni2	0.02	0.03
25	239405	Rspo2	0.02	0.02
26	11302	Aatk	0.02	0.02
27	26874	Abcd2	0.02	0.02
28	66447	Mgst3	0.03	0.03
29	12766	Cxcr3	0.03	0.02
30	16170	Il16	0.03	0.03
31	66355	Gmpr	0.03	0.01
32	97998	Deptor	0.03	0.02
33	14431	Gamt	0.03	0.02
34	12442	Ccnb2	0.03	0.01
35	269642	Nat8l	0.03	0.03
36	14456	Gas6	0.03	0.02
37	217262	Abca9	0.03	0.02
38	213573	Cracr2b	0.03	0.02
39	22402	Wisp1	0.03	0.03
40	233552	Gdpd5	0.03	0.02
41	213068	Tmem71	0.03	0.03
42	78771	Mctp1	0.03	0.03
43	16970	Lrmp	0.03	0.03
44	66425	Pcp4l1	0.03	0.03
45	240168	Rasgrp3	0.03	0.02
46	321019	Gpr183	0.03	0.03
47	381101	Dnph1	0.03	0.06
48	12925	Crip1	0.03	0.04
49	217946	Cdca7l	0.03	0.02
50	15985	Cd79b	0.03	0.04

Supplemental Table 2: *List of top genes dysregulated by IL4/IL13.*

Fold Change: up > 2 fold (red)
Fold Change: down < 0.5 fold (green)

S. No.	Gene ID	Gene Name	WT IL4/IL13 vs WT-Mφ	$Notch1^{+/-}$ IL4/IL13 vs $Notch1^{+/-}$ Mφ
1	104183	*Chil4*	661.44	1189.90
2	12655	*Chil3*	504.92	1049.84
3	58205	*Pdcd1lg2*	360.54	434.36
4	57262	*Retnla*	299.69	311.49
5	93726	*Rnase2a*	141.66	93.64
6	19662	*Rbp4*	129.07	295.54
7	12700	*Cish*	110.57	134.17
8	327766	*Tmem26*	106.27	101.68
9	229687	*Chil5*	87.30	130.85
10	229688	*Chil6*	62.31	98.82
11	16165	*Il13ra2*	52.81	61.25
12	20292	*Ccl11*	37.46	45.23
13	245126	*Tarm1*	31.21	25.26
14	17207	*Mcf2l*	22.45	27.39
15	16364	*Irf4*	20.35	17.29
16	215446	*Entpd3*	18.79	4.97
17	11846	*Arg1*	18.68	17.00
18	319930	*Ceacam19*	17.24	16.22
19	107221	*Ffar4*	16.54	17.14
20	11988	*Slc7a2*	15.83	14.05
21	13011	*Cst7*	15.76	17.93
22	245533	*Awat1*	15.74	17.64
23	327957	*Scimp*	14.35	7.89
24	70166	*Lipn*	13.46	7.88
25	16625	*Serpina3c*	13.45	11.72
26	14254	*Flt1*	13.21	10.58
27	12703	*Socs1*	13.18	18.46
28	20299	*Ccl22*	13.08	13.81
29	104886	*Rab15*	12.54	12.63
30	12845	*Comp*	12.09	6.01
31	19224	*Ptgs1*	12.07	10.88
32	76477	*Pcolce2*	12.04	12.22
33	240327	*Gm4951*	11.76	9.62
34	406217	*Bex4*	11.50	14.89
35	56221	*Ccl24*	11.26	4.68
36	216864	*Mgl2*	10.91	14.95
37	19716	*Bex1*	10.77	15.12
38	110074	*Dut*	10.33	7.03
39	233079	*Ffar2*	9.96	15.32
40	108956	*Apol7c*	9.91	10.09
41	665033	*Col6a5*	9.46	6.96
42	380728	*Kcnh4*	9.26	7.80
43	101772	*Ano1*	9.24	2.17
44	71111	*Gpr39*	8.99	5.94
45	20293	*Ccl12*	8.77	4.79
46	15891	*Ibsp*	8.43	13.07
47	15220	*Foxq1*	8.42	13.01
48	15558	*Htr2a*	8.05	4.45
49	15937	*Ier3*	7.92	7.39
50	67573	*Loxl4*	7.77	2.01

S. No.	Gene ID	Gene Name	WT IL4/IL13 vs WT-Mφ	$Notch1^{+/-}$ IL4/IL13 vs $Notch1^{+/-}$ Mφ
1	229499	*Fcrl1*	0.03	0.06
3	105349	*Akr1c18*	0.07	0.12
4	17392	*Mmp3*	0.07	0.09
5	12766	*Cxcr3*	0.08	0.06
6	56811	*Dkk2*	0.08	0.07
7	14985	*H2-M10.1*	0.09	1.00
8	66425	*Pcp4l1*	0.10	0.06
9	11670	*Aldh3a1*	0.10	0.16
10	14191	*Fgr*	0.11	0.30
11	23890	*Gpr34*	0.13	0.15
12	12273	*C5ar1*	0.13	0.13
13	57442	*Kcne3*	0.13	0.26
14	56620	*Clec4n*	0.14	0.10
15	14788	*Gpr162*	0.14	0.15
16	12161	*Bmp6*	0.14	0.11
17	232413	*Clec12a*	0.14	0.10
18	106952	*Arap3*	0.15	0.13
19	18591	*Pdgfb*	0.15	0.19
20	70261	*Chp2*	0.16	0.25
21	14451	*Gas1*	0.16	0.39
22	242425	*Gabbr2*	0.16	0.22
23	50723	*Icosl*	0.16	0.17
24	53945	*Slc40a1*	0.17	0.14
25	11522	*Adh1*	0.17	0.94
26	71738	*Mamdc2*	0.17	0.29
27	170757	*Adgrl4*	0.19	0.46
28	20513	*Slc1a6*	0.19	0.08
29	108723	*Card11*	0.20	0.44
30	14585	*Gfra1*	0.20	0.30
31	67888	*Tmem100*	0.20	0.31
32	74145	*F13a1*	0.20	0.14
33	723814	*Mir223*	0.21	0.54
34	75581	*Yipf7*	0.21	0.20
35	117590	*Asb10*	0.21	0.25
36	16010	*Igfbp4*	0.22	0.14
37	16633	*Klra2*	0.22	0.29
38	20303	*Ccl4*	0.22	0.25
39	75604	*Tm4sf5*	0.22	0.44
40	216799	*Nlrp3*	0.22	0.12
41	12504	*Cd4*	0.22	0.36
42	78303	*Hist3h2ba*	0.23	0.95
43	73708	*Dppa3*	0.23	0.41
44	12721	*Coro1a*	0.23	0.23
45	17384	*Mmp10*	0.24	0.58
46	320712	*Abi3bp*	0.24	0.27
47	212398	*Frat2*	0.24	0.31
48	56198	*Heyl*	0.24	0.77
49	11629	*Aif1*	0.24	0.20
50	11784	*Apba2*	0.24	0.26

Supplemental Table 3: List of primer sequences used in quantitative real-time reverse transcriptase PCR studies.

Gene Symbol	Forward Primer	Reverse Primer
Rpl13a	5'- TCC CTG CTG CTC TCA AGG-3'	5'- GCC CCA GGT AAG CAA ACT T-3'
Notch1	5'-CCG TTA CAT GCA GCA GTT TC-3'	5'-AGC CAG GAT CAG TGG AGT TG-3'
Dll4	5'-GAC CTG CGG CCA GAG ACT T-3'	5'-GAG CCT TGG ATG ATG ATT TGG-3'
Jag1	5'-GGC TTC TCA CTC AGG CAT GAT A-3'	5'-GTG GGC AAT CCC TGT GTT TT-3'
RbpjK	5'-AAG AGT CTC AAC CCT GTG CG-3'	5'-CAC TGT TGT GAA CTG GCG TG-3'
Hey1	5'-CGC GGA CGA GAA TGG AAA CT-3'	5'-TCT CGA TGA TGC CTC TCC GT-3'
Hey2	5'-TGG GGA GCG AGA ACA ATT AC-3'	5'-CCT CGC CAC TTC TGT TAA GC-3'
HeyL	5'-GGA ACA ACA GAG AAT GAA CCA ACC-3'	5'-TCT TGA CTT TGC CAG GGA TTA GC-3'
Hes1	5'-CCC CAG CCA GTG TCA ACA C-3'	5'-TGT GCT CAG AGG CCG TCT T-3'
Casp4	5'-CAA TGG CCG TAC ACG AAA GG-3'	5'-GCC CCA TAC CTC AGT GAG AGA T-3'
Fmod	5'-GTC CAC CTA CTA CGA CCC CT-3'	5'-GAC AGT CGC ATT CTT GGG GA-3'
Alpl	5'-TGG TAT GGG CGT CTC CAC AGT AAC C-3'	5'-CTT GGA GAG GGC CAC AAA GG-3'
Col1a1	5'-GAG CGG AGA GTA CTG GAT CG-3'	5'-GTT CGG GCT GAT GTA CCA GT-3'
Igfbp2	5'-CTG CAC ATC CCC AAC TGT GA-3'	5'-CGC TGT CCG TTC AGA GAC AT-3'
Lum	5'-TCG AGC TTG ATC TCT CCT AT-3'	5'-TGG TCC CAG GAT CTT ACA GAA-3'

Figure-5 Hans

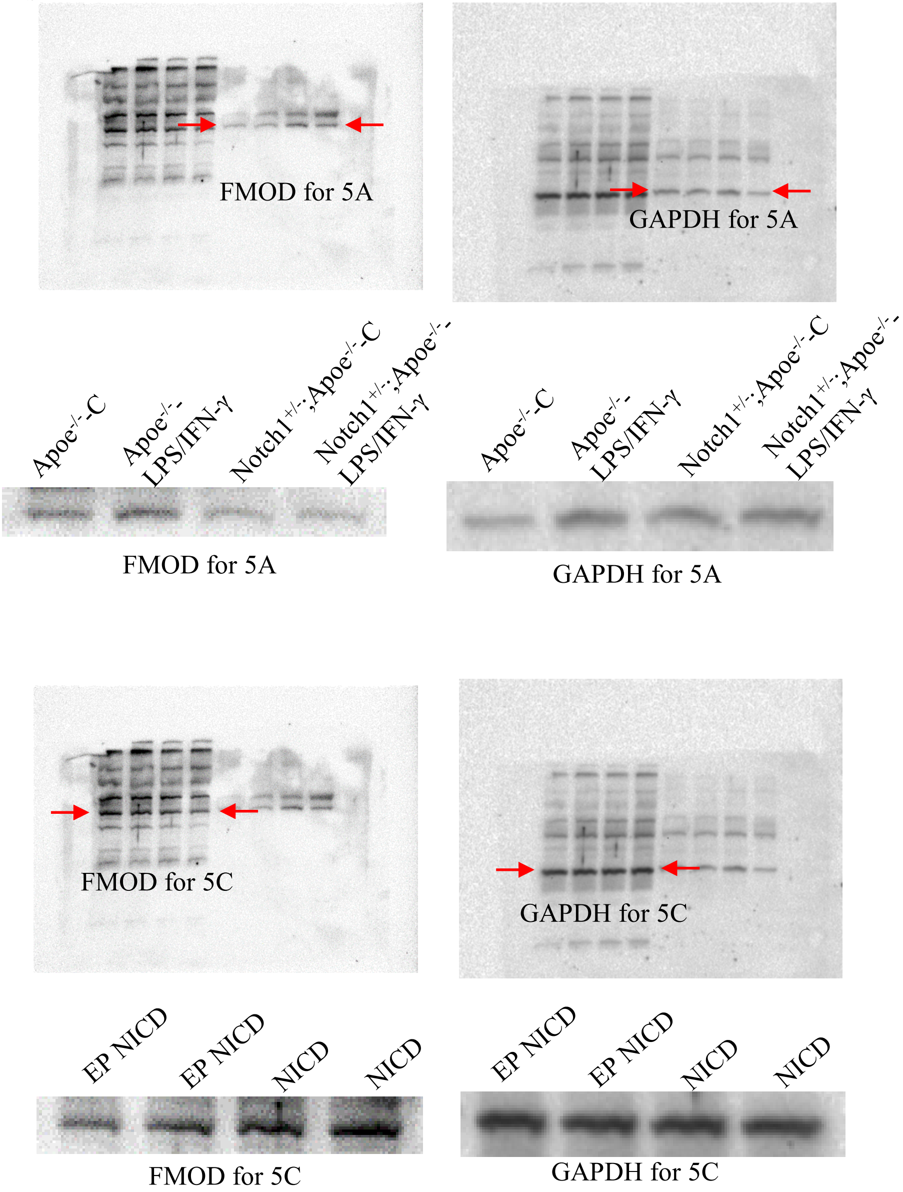

Figure-6 Hans

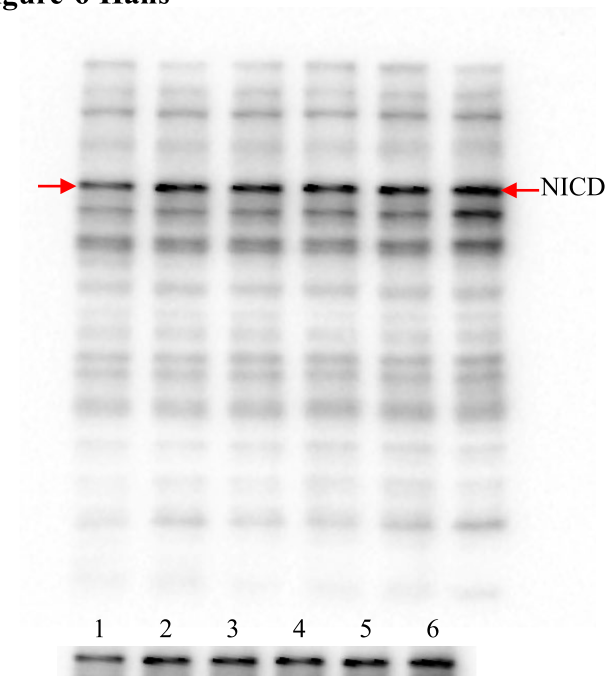

NICD for 6A

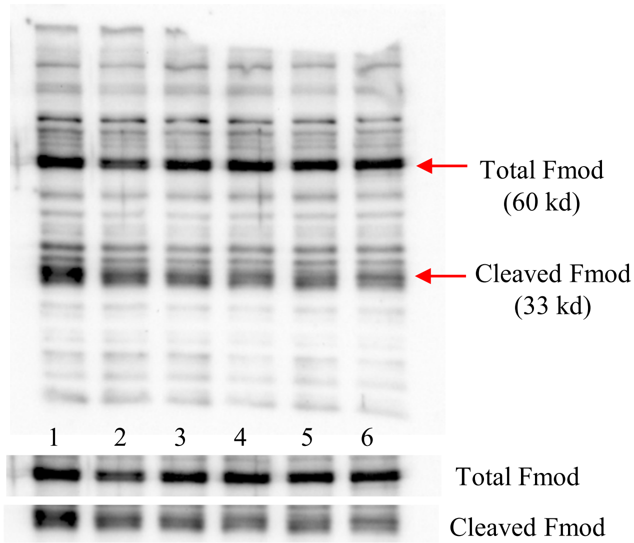

Fmod Total and cleaved for 6A

1. Control
2. LPS/IFN-γ -5 ng/ml
3. LPS/IFN-γ -10 ng/ml
4. LPS/IFN-γ -25 ng/ml
5. LPS/IFN-γ -50 ng/ml
6. LPS/IFN-γ -100 ng/ml

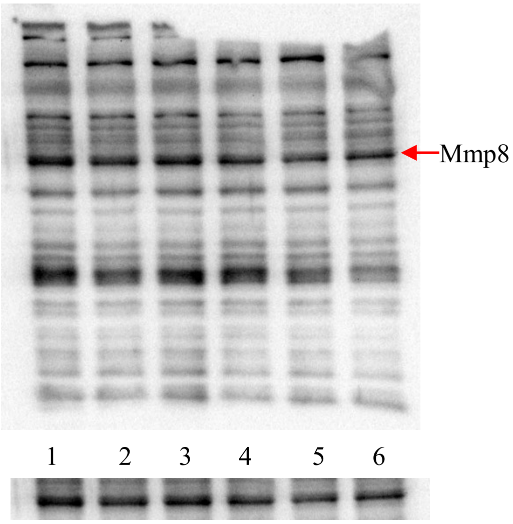

Mmp8 for 6A

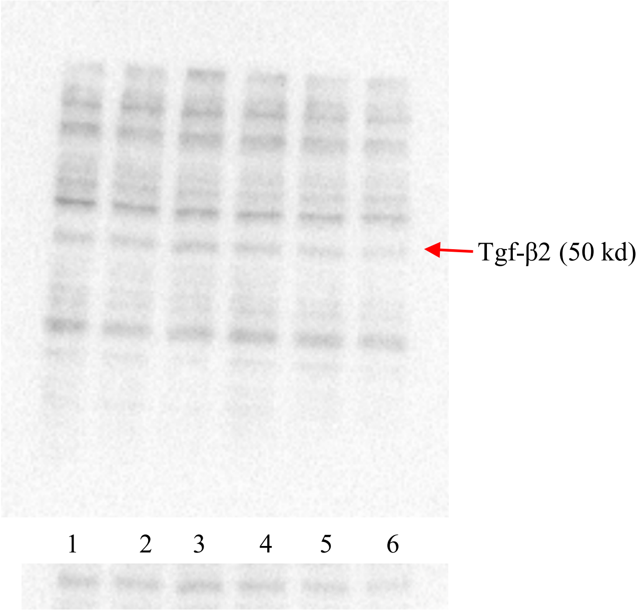

Tgf-β2 for 6A

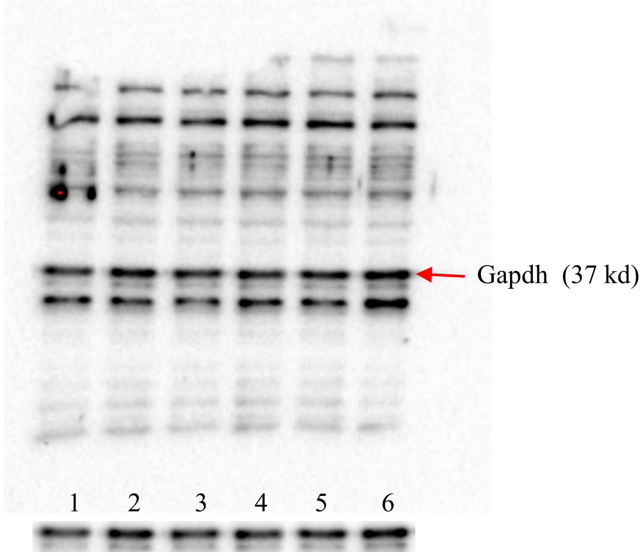

GAPDH for 6A

Figure-7 Hans

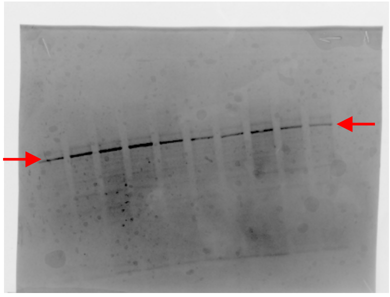

Tgf-β2 for 7G

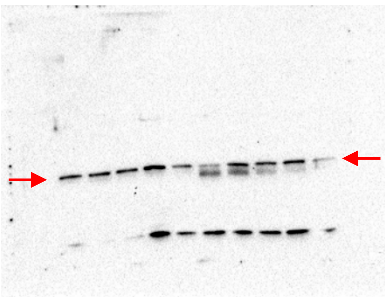

CBB for 7G

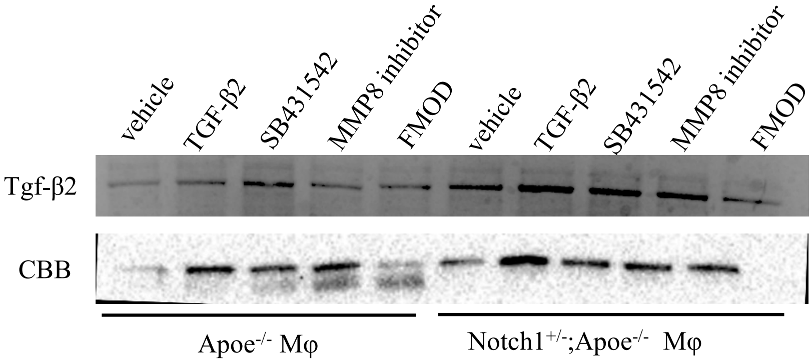

Tgf-β2 and CBB for 7G

Supplemental Figure-3 Hans

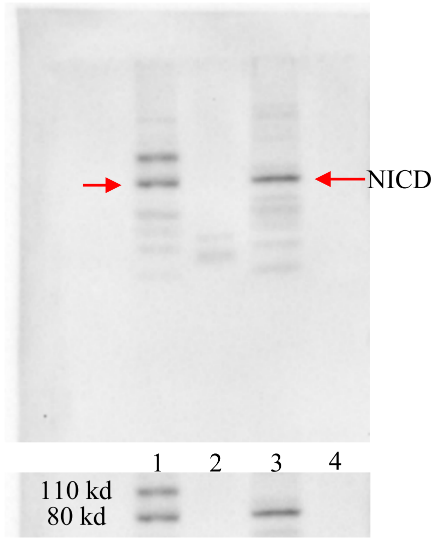

NICD for S3G

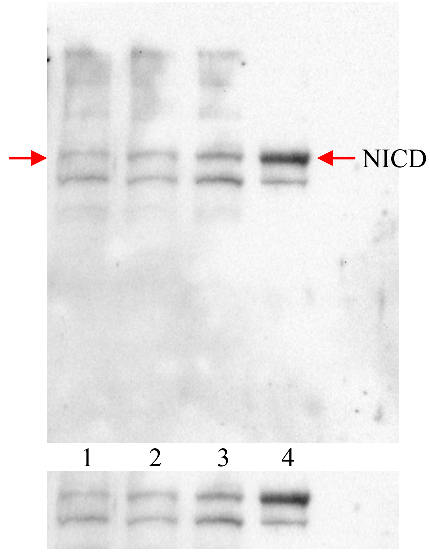

NICD for S3H

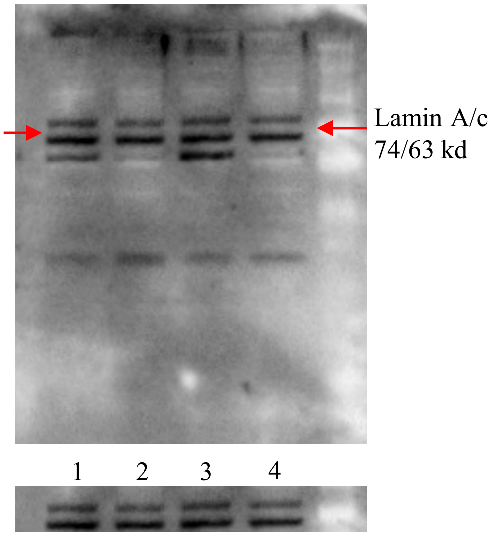

LaminA/c for S3G

1. Control
2. Notch1 siRNA
3. LPS
4. LPS+ Notch1 siRNA

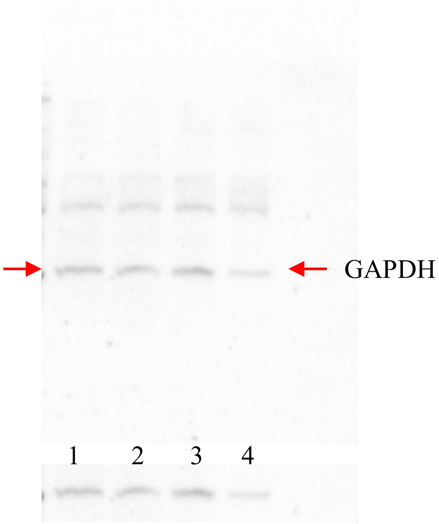

GAPDH for S3H

1. Empty plasmid
2. Empty plasmid
3. NICD
4. NICD
